# Meta-analysis of hybrid immunity to mitigate the risk of Omicron variant reinfection

**DOI:** 10.3389/fpubh.2024.1457266

**Published:** 2024-08-26

**Authors:** Huiling Zheng, Shenggen Wu, Wu Chen, Shaojian Cai, Meirong Zhan, Cailin Chen, Jiawei Lin, Zhonghang Xie, Jianming Ou, Wenjing Ye

**Affiliations:** ^1^Institute of Emergency Response and Epidemic Management, Fujian Provincial Center for Disease Control and Prevention, Fuzhou, China; ^2^School of Public Health, Fujian Medical University, Fuzhou, China

**Keywords:** hybrid immunity, Omicron reinfection, meta-analysis, SARS-CoV-2, prevention and control of infectious diseases

## Abstract

**Background:**

Hybrid immunity (a combination of natural and vaccine-induced immunity) provides additional immune protection against the coronavirus disease 2019 (COVID-19) reinfection. Today, people are commonly infected and vaccinated; hence, hybrid immunity is the norm. However, the mitigation of the risk of Omicron variant reinfection by hybrid immunity and the durability of its protection remain uncertain. This meta-analysis aims to explore hybrid immunity to mitigate the risk of Omicron variant reinfection and its protective durability to provide a new evidence-based basis for the development and optimization of immunization strategies and improve the public’s awareness and participation in COVID-19 vaccination, especially in vulnerable and at-risk populations.

**Methods:**

Embase, PubMed, Web of Science, Chinese National Knowledge Infrastructure, and Wanfang databases were searched for publicly available literature up to 10 June 2024. Two researchers independently completed the data extraction and risk of bias assessment and cross-checked each other. The Newcastle-Ottawa Scale assessed the risk of bias in included cohort and case–control studies, while criteria recommended by the Agency for Health Care Research and Quality (AHRQ) evaluated cross-sectional studies. The extracted data were synthesized in an Excel spreadsheet according to the predefined items to be collected. The outcome was Omicron variant reinfection, reported as an Odds Ratio (OR) with its 95% confidence interval (CI) and Protective Effectiveness (PE) with 95% CI. The data were pooled using a random- or fixed-effects model based on the *I^2^* test. The Preferred Reporting Items for Systematic Reviews and Meta-Analyses guidelines were followed.

**Results:**

Thirty-three articles were included. Compared with the natural immunity group, the hybrid immunity (booster vaccination) group had the highest level of mitigation in the risk of reinfection (OR = 0.43, 95% CI:0.34–0.56), followed by the complete vaccination group (OR = 0.58, 95% CI:0.45–0.74), and lastly the incomplete vaccination group (OR = 0.64, 95% CI:0.44–0.93). Compared with the complete vaccination-only group, the hybrid immunity (complete vaccination) group mitigated the risk of reinfection by 65% (OR = 0.35, 95% CI:0.27–0.46), and the hybrid immunity (booster vaccination) group mitigated the risk of reinfection by an additional 29% (OR = 0.71, 95% CI:0.61–0.84) compared with the hybrid immunity (complete vaccination) group. The effectiveness of hybrid immunity (incomplete vaccination) in mitigating the risk of reinfection was 37.88% (95% CI, 28.88–46.89%) within 270–364 days, and decreased to 33.23%% (95% CI, 23.80–42.66%) within 365–639 days; whereas, the effectiveness after complete vaccination was 54.36% (95% CI, 50.82–57.90%) within 270–364 days, and the effectiveness of booster vaccination was 73.49% (95% CI, 68.95–78.04%) within 90–119 days.

**Conclusion:**

Hybrid immunity was significantly more protective than natural or vaccination-induced immunity, and booster doses were associated with enhanced protection against Omicron. Although its protective effects waned over time, vaccination remains a crucial measure for controlling COVID-19.

**Systematic review registration:**

https://www.crd.york.ac.uk/PROSPERO/, identifier, CRD42024539682.

## 1 Introduction

The emergence and rapid spread of coronavirus disease 2019 (COVID-19) caused by Severe Acute Respiratory Syndrome Coronavirus 2 (SARS-CoV-2), a potentially fatal disease, rapidly led to a global public health crisis (1). SARS-CoV-2 enters human cells with the help of the S glycoprotein, a trimeric transmembrane protein, by interacting with the ACE2 receptor on the host cell’s surface (2). SARS-CoV-2 has continuously mutated, with at least 10 significant variants emerging, known as Variants of Concern(VOCs) (2). These VOCs prompted stricter lockdown measures and travel restrictions globally (2, 3). Despite these efforts, reinfections have occurred since the first wave of COVID-19 (3). SARS-CoV-2 reinfection refers to an individual who recovered from infection with a particular SARS-CoV-2 variant and then became infected with a new or previous variant at a certain time interval (4). The Omicron variant, first detected in South Africa in November 2021, has more mutation sites than other variants, particularly in the receptor-binding domain of the spike protein (S) (4, 5). It can effectively bind to human ACE2 to enter host cells, facilitating faster virus replication and increased transmissibility (1, 5). These mutations can evade antibodies produced after natural infection, leading to more reinfections (3). During the Omicron epidemic, the reinfection rate increased significantly (4, 6). Although most infections are mild or asymptomatic, reinfection poses an elevated risk of mortality and long-term sequelae, regardless of the degree of infection, and the cumulative risk and disease burden increase with the number of infections (7). Despite advancements in vaccine technology technologies and mass administration of booster doses, Omicron continues to emerge in its new and more potent subvariants, resulting in delayed vaccination campaigns and immunization responses (2).

The occurrence of reinfection is closely related to the level of human immunity, and the immune memory of the body against SARS-CoV-2 can be induced through natural infection or vaccination (8). The global surge in Omicron variants has resulted in many individuals developing hybrid immunity (a combination of natural and vaccine-induced immunity) (9), which reflects the actual immune status of the population and their ability to cope with viral infections. Hybrid immunity has gained attention because of its potential to provide additional immune protection. However, the continually evolving Omicron variant poses a significant threat to antibody therapies and the currently authorized COVID-19 vaccines due to its profound immune evasion potential (10). This necessitates reassessing the protective effect of hybrid immunity against Omicron reinfection to tailor vaccination guidance optimally (9). Our extensive search revealed that very few systematic reviews (9) included data related to hybrid immunity and the Omicron variant and did not include many recent studies. A previous review up to July 2022 showed a stronger protective effect of hybrid immunity compared to natural immunity but focused mainly on Delta, Omicron, and other variants like Gamma, with fewer studies on Omicron reinfections (11). A meta-analysis by Joshua Nealon et al. (12) up to December 31, 2022, assessed the effectiveness of the COVID-19 monovalent booster vaccine and hybrid immunity against Omicron but did not clarify the efficacy of different vaccine dosages. Furthermore, some systematic reviews have evaluated the preventive effect of vaccines against the Omicron variant and defined the hybrid vaccination of different technical routes of vaccines as hybrid immunity (13–18). The efficacy of vaccination is related to vaccination dosage and vaccination intervals (13). However, these studies focused on only one of two aspects and did not determine whether it was primary or reinfection with Omicron (13–18). Given the widespread infection and vaccination today, it is challenging to distinguish between the effectiveness of vaccines and natural infection, potentially leading to public misconceptions and vaccine hesitancy that it is safe to have been infected with COVID-19 and to have completed the primary vaccination. For example, healthcare workers, a priority group for vaccination, have shown significant hesitancy to receive a second booster dose in various countries (19, 20). To sum up, the effect of hybrid immunity on mitigating Omicron reinfection risk and its protective durability has not been fully determined. The World Health Organization believes more data are needed to quantify these effects accurately (21). Moreover, incorporating vaccine knowledge and new evidence into routine health education and procedures to raise confidence and reduce complacency may be effective and feasible for promoting vaccination and implementing future vaccination programs (19). Therefore, the outstanding advantage of our meta-analysis over previous studies is not only that the search time was sufficiently long to represent the epidemiological period of Omicron but also that it comprehensively assessed the mitigation of the risk of reinfection and the durability of protection by hybrid immunity against the Omicron variant by setting up all possible control groups, including vaccination only, natural infection only, and different vaccination dosages, aiming to provide new scientific evidence for the formulation and optimization of immunization strategies and to improve the public’s awareness and participation in COVID-19 vaccination for better promotion of the future immunization planning.

## 2 Methods

### 2.1 Review registration and design

This study was conducted in strict adherence to the Preferred Reporting Items for Systematic Reviews and Meta-Analyses guidelines (22) (Supplementary Table S1) and was prospectively registered in the International Prospective Register of Systematic Reviews (PROSPERO) under registration number CRD42024539682.

### 2.2 Data sources and searches

We searched Embase, PubMed, Web of Science, Chinese National Knowledge Infrastructure (CNKI), and Wanfang databases for publicly available literature from their inception to 10 June 2024. The search keywords focused on SARS-CoV-2/COVID-19, Reinfection, Hybrid Immunity, Prior Infection, Initial Infection, Natural Immunity, and Vaccination. Boolean logic operators were used to link the search terms. Literature management was performed using Endnote X9 software. The detailed search procedures for each database were provided in Supplementary Data Sheet S1.

### 2.3 Study inclusion and exclusion criteria

Inclusion criteria: (1) studies included at least one group of people with natural immunity against SARS-CoV-2; (2) according to the different types of immuniszation modalities, the analysis was carried out in the following groups: i) the exposed group was hybrid immunity, while the control group was only natural immunity; ii) the exposed group was hybrid immunity, while the control group was only vaccine immunity; iii) the exposed group was hybrid immunity (booster vaccination), while the control group was hybrid immunity (complete vaccination); the exposed group was hybrid immunity (booster vaccination / complete vaccination), while the control group was hybrid immunity (incomplete vaccination); iv) the exposed group was hybrid immunity, while the control group was in a non-immune state; (3) the study outcome was any confirmed Omicron reinfections, including mild, symptomatic, and asymptomatic reinfections; (4) the literature has clearly stated the definition of SARS-CoV-2 reinfection and the time interval between the first or prior infection and reinfection to exclude patients with recurrent positivity; (5) the type of study was observational; (6) the literature provided raw data that could be converted to Odds Ratio (OR); (7) the estimated Protective Effectiveness (PE) and 95% confidence interval (CI) of hybrid immunity in mitigating the risk of Omicron reinfection over time; (8) no language restrictions.

Exclusion criteria: (1) duplicate publications, literature without sources; (2) literature unrelated to the current research topic(e.g., antibody, clinical cases with a history of specific diseases, etc); (3) reinfection with non-Omicron variants and unspecified types of reinfection variants; (4) case reports or case series analysis, meta-analyses, systematic reviews, animal experiments, conference papers, etc.; (5) the time interval between primary or prior infection and reinfection was not clearly stated and data could not be extracted.

### 2.4 Data extraction and quality assessment

Two researchers independently completed the literature search, screening, data extraction, and risk of bias assessment, and cross-checked each other. Any disagreement was discussed and resolved with the third researcher. The Newcastle-Ottawa Scale assessed the risk of bias in included cohort and case–control studies, while criteria recommended by the Agency for Health Care Research and Quality (AHRQ) of the United States evaluated cross-sectional studies (23) (Supplementary Tables S2–S4). Data extraction includes (1) the first author, year of publication, study area, and study design; (2) vaccination status, vaccine type, Omicron subvariants, the time between initial or prior infection and reinfection, the time between reinfection and last vaccination; (3) number of exposed and control groups, PE and its 95% CI.

To evaluate the validity and robustness of our meta-analysis, two well-established techniques were employed: the Assessment of Multiple Systematic Reviews (AMSTAR) and the Quality of Reporting of Meta-analyses (QUOROM) checklist (24) (Supplementary Tables S5, S6). Additionally, we undertook an extensive search to identify systematic reviews and meta-analyses potentially related to our topic. Embase, Web of Science, and PubMed were examined from November 1, 2020, to July 14, 2024, with the keywords including Omicron, previous infection, vaccine, hybrid immunity, meta-analysis, etc. (Supplementary Table S7). We used the GROOVE (Graphical Representation of Overlap for Overviews) tool to complete pairwise intersection heat maps, corrected covered area (CCA), and a citation matrix of evidence to evaluate the primary study overlap (24) (Supplementary Table S8).

### 2.5 Data synthesis

Based on synthesized data, the following factors were defined: (1) hybrid immunity was natural immunity induced by previous infection combined with immunity induced by vaccination. (2) Vaccination status was divided into: i) unvaccinated, defined as not vaccinated with the COVID-19 vaccine; ii) incomplete vaccination, defined as failure to complete the primary vaccination program; iii) complete vaccination, defined as the completion of the primary vaccination program; iv) booster vaccination, defined as the booster vaccination after the completion of the primary vaccination. (3) It is important to recognize that the vaccination protocols may be adjusted in light of the epidemic prevention and control situation, research progress, population selection, and regional specificities. However, we acknowledge that some of the studies included in our analysis did not explicitly state the type of vaccine administered. To ensure a comprehensive and representative sample of research and to align our findings with real-world vaccination practices, we adopted an inclusive approach to the vaccination protocols considered in our study. This included a variety of formulations such as adenoviral vector vaccines, mRNA vaccines, and inactivated vaccines. When studies involved the mRNA vaccines (e.g., BNT162b2, mRNA-1273) or inactivated vaccines (e.g., CoronaVac), the primary vaccination was defined as complete upon receiving two doses (25), with a requirement in the majority of studies that at least 14 days had elapsed since the second dose (7). Reinfection within 14 days after the second dose and receiving only one dose were regarded as incomplete primary vaccination (7). Conversely, for studies using adenoviral vector vaccines (e.g., ChAdOx1-S, Ad26.COV2.S), primary vaccination was typically achieved with a single dose, provided that more than 14 days had passed since administration (9). Booster immunization could either be homologous or heterologous booster vaccination, reflecting the diverse approaches to enhancing immunity post-primary vaccination.

The examination of protection durability against Omicron reinfection within the hybrid immunity group, categorized by individuals’ vaccination status following prior infection, was divided into three time frames: time since last incomplete vaccination, time since complete vaccination, and time since booster vaccination (median time/follow-up time range).

### 2.6 Data analysis

The “meta” package (version 6.5–0) in R (version 4.2.3; R Core Team, 2023) was used for data analysis. The effect estimates were the pooled OR and 95% CI, as well as the pooled PE and 95% CI. The *I^2^* test was used to assess heterogeneity among studies, with *I^2^* ≥ 50% and *p* ≤ 0.05 indicating significant heterogeneity and warranting the use of random-effects models; otherwise, fixed-effects models were selected. Sensitivity analysis involved recalculating the pooled effect estimates by sequentially removing each study and comparing them with the original estimates to assess the robustness of the results. Egger’s test and funnel plot were used to evaluate publication bias when the number of original studies was ≥10. If publication bias was detected, the nonparametric trim-and-fill method was used to evaluate its impact on the results. The significance level was set at α = 0.05.

## 3 Results

### 3.1 Basic characteristics and quality of included studies

**Figure 1:**
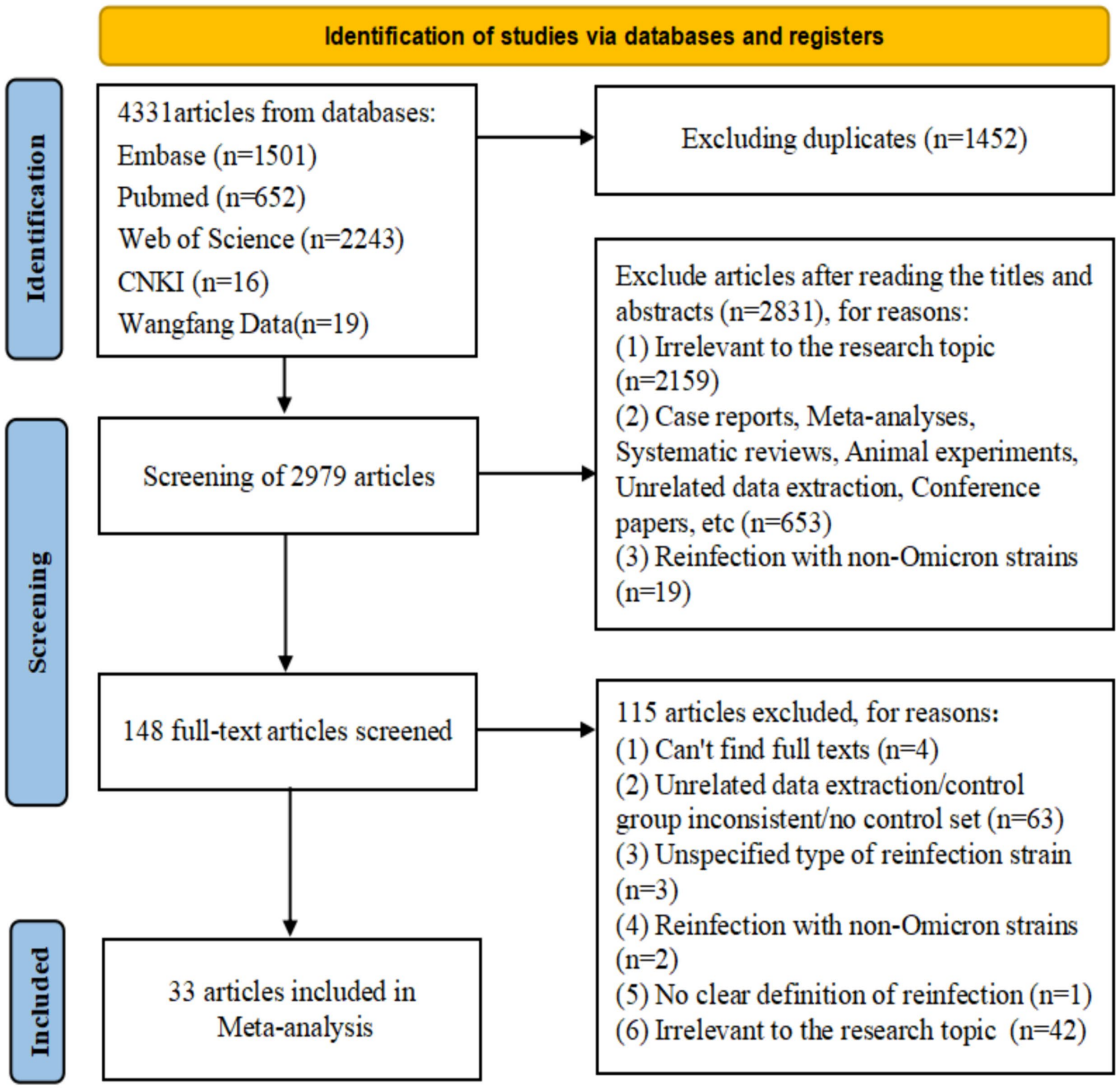
Preferred Reporting Items for Systematic Reviews and Meta-Analyses (PRISMA model) flow map of article selection Supplementary Table S9.

After screening and removing duplicates, 33 studies from 16 countries were included (25–57), of which 12 were from Asia (25, 30–33, 35, 42–44, 50, 53, 57), 10 from Europe (27, 29, 36, 39–41, 45, 46, 52, 56), 8 from North America (26, 28, 37, 38, 47, 49, 51, 54), 2 from South America (34, 48), and 1 from Oceania (55). There were 13cohort (25, 27–30, 34, 38, 39, 41, 43, 45, 55, 56), 14 case–control (26, 31, 36, 37, 40, 46–52, 54, 57), and 6 cross-sectional studies (32, 33, 35, 42, 44, 53). In terms of quality, 31 articles (25–38, 40–52, 54–57) were considered high quality, whereas two articles (39, 53) were of medium quality (Supplementary Tables S2–S4). Most studies have performed a risk analysis of hybrid immunity on Omicron variant reinfection (30–39, 41–45, 50–53, 55, 56). Regarding the protective durability of hybrid immunity in mitigating Omicron variant reinfection, 14 articles (25–29, 36, 40, 46–51, 54) focused on hybrid immunity with incomplete vaccination, seven articles (36, 40, 46, 48–50, 57) on hybrid immunity with complete vaccination, and seven articles (36, 46–51) on hybrid immunity with booster vaccination. The basic characteristics and quality of the included studies were presented in detail in the Supplementary Table S10 (Figure 1; Supplementary Tables S9, S10).

### 3.2 The assessment of the study quality of this meta-analysis and the overlap of primary studies

The total AMSTAR score of this study was 10 points, and the full score of the scale was 11 points, indicating that the overall methodological quality of our meta-analysis was relatively good (Supplementary Table S5). It is worth mentioning the grey literature, that is, studies with positive results are willing to be submitted to English magazines, while studies with negative results are very likely to be put aside. Language is an important factor affecting search bias. Although our study conducted a comprehensive literature search without considering language restrictions, the studies that ultimately met the inclusion criteria included both Chinese and English literature, including preprints. However, there may still potentially exist undiscovered grey literature. Similarly, The QUOROM score was 16, whose maximum might be 18, implying that the reporting quality of our study was at a medium to high level (Supplementary Table S6).

Applying our defined inclusion and exclusion criteria, we identified three meta-analyses closely related to our topic (Supplementary Table S7). One of these (12) was excluded since the included index studies could not be extracted, and the influence of hybrid immunity on the reinfection risk of the Omicron variant was a secondary aspect of this study. The remaining two studies (9, 11) presented a moderate overlap (CCA = 9.5%) (Supplementary Table S8). Considering the paucity of published meta-analyses and the relatively brief search period, both concluding in July 2022, it was infeasible to ensure a comprehensive overview of the research domain. In actuality, a significant number of original studies on hybrid immunity and Omicron were published in 2023 and 2024, highlighting the need for an updated review. Simultaneously, the homogeneity and correlation between the two meta-analyses were low, and research gaps persisted. It was inadequate to support our second-order meta-analysis on the topic of hybrid immunity mitigating the reinfection risk and protection durability of Omicron. Therefore, our meta directly aggregated data from the index studies and did not undertake a direct summary of the meta-analysis. The exclusion criteria of the study encompassed meta-analyses and systematic reviews. Additionally, the search time of our study concluded on June 10, 2024, covering the two meta-analyses. Hence, some similar index studies would be included, resulting in a CCA score higher than 15% (21.82%) between this study and the two studies. It was also associated with the inclusion of more studies published in 2023 and 2024 in this meta-analysis. When calculating the CCA, structural missingness (or structural zeros) was taken into consideration. That is, the systematic reviews published in 2022 could not incorporate the primary studies published in 2023 (24). The matrix contained more counts of structural zeros, and the adjusted CCA value increased after adjusting for the structural zeros (Supplementary Table S8). Therefore, our meta-analysis has undertaken an extensive search, aggregated data from the index studies, and covered the Omicron epidemic, minimizing information omission.

### 3.3 Risk analysis of hybrid immunity for the Omicron variant reinfection

**Figure 2:**
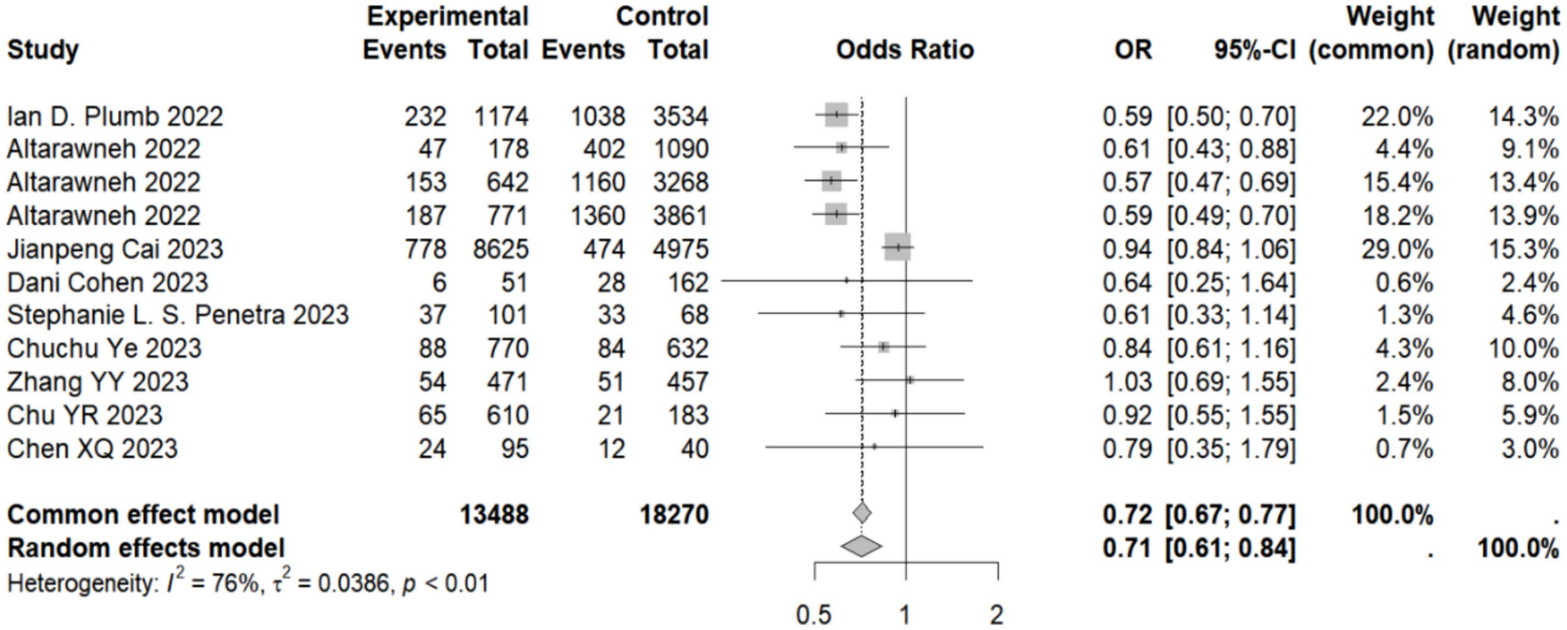
Forest plot of meta-analysis of the effect of hybrid immunity with booster vaccination on the risk of reinfection with Omicron variants (compared to the hybrid immunity with complete vaccination group). Forest plots of all the analysis groups were presented in the Supplementary Table S11.

Our meta-analysis showed that compared with the natural immunity group, the pooled OR was 0.64 (95% CI, 0.44–0.93), 0.58 (95% CI, 0.45–0.74), and 0.43 (95%CI, 0.34–0.56) in the hybrid immunity (incomplete vaccination), hybrid immunity (complete vaccination) and hybrid immunity (booster vaccination) groups, respectively. Compared with the complete vaccination group, the pooled OR for the hybrid immunity (complete vaccination) group was 0.35 (95% CI, 0.27–0.46), and compared with the booster vaccination group, the pooled OR for the hybrid immunity (booster vaccination) group was 0.29 (95% CI, 0.17–0.47). The pooled OR was 0.69 (95% CI, 0.55–0.87) in the hybrid immunity (complete vaccination) group and 0.50 (95% CI, 0.36–0.69) in the hybrid immunity (booster vaccination) group compared with the hybrid immunity (incomplete vaccination) group. Compared with the hybrid immunity (complete vaccination) group, the OR of the hybrid immunity (booster vaccination) group was 0.71 (95% CI, 0.61–0.84) (Figure 2; Table 1). Forest plots were presented in the Supplementary Table S11.

**Table 1:** Risk analysis of hybrid immunity against reinfection with Omicron variant.

Exposure group	Control group	Number of articles	Number of studies	Exposure group		Control group		Pooled OR estimate and 95% CI	*p*	*I^2^* (%)	*p*	Effect model	*p* for Egger’s test
				The number of reinfections	Total number	The number of reinfections	Total number						
Hybrid immunity (incomplete vaccination)	Natural immunity	10	11	17,285	1,782,524	187,528	19,535,373	0.64 (0.44–0.93)	0.027	97.0	<0.001	Random	0.962
Hybrid immunity (complete vaccination)	Natural immunity	13	25	53,020	5,044,281	231,588	19,739,066	0.58 (0.45–0.74)	<0.001	99.0	<0.001	Random	0.392
Hybrid immunity (booster vaccination)	Natural immunity	13	18	28,079	8,769,132	191,164	19,518,535	0.43 (0.34–0.56)	<0.001	97.0	<0.001	Random	0.126
Hybrid immunity (complete vaccination)	Complete vaccination	3	7	5,280	40,416	67,011	327,551	0.35 (0.27–0.46)	<0.001	97.0	<0.001	Random	
Hybrid immunity (booster vaccination)	Booster vaccination	4	14	3,489	104,802	159,244	1,252,783	0.29(0.17–0.47)	<0.001	99.0	<0.001	Random	0.303
Hybrid immunity (booster vaccination)	Hybrid immunity (complete vaccination)	9	11	1,671	13,488	4,663	18,270	0.71 (0.61–0.84)	<0.001	76.0	<0.001	Random	0.806
Hybrid immunity (booster vaccination)	Hybrid immunity (incomplete vaccination)	9	9	23,315	8,701,929	1827	165,726	0.50 (0.36–0.69)	<0.001	92.0	<0.001	Random	
Hybrid immunity (complete vaccination)	Hybrid immunity (incomplete vaccination)	9	9	23,404	4,714,338	2,519	173,648	0.69 (0.55–0.87)	<0.001	93.0	<0.001	Random	

### 3.4 Analysis on the durability of protection against Omicron reinfection with hybrid immunity (incomplete vaccination)

Hybrid immunity (incomplete vaccination) within 60 days can mitigate the risk of Omicron reinfection by 66.76% (95% CI, 58.20–75.32%). However, the protective effectiveness decreased to 64.25% (95% CI, 53.72–74.77%) between 60 and 89 days, further decreasing to 59.50% (95% CI, 55.56–63.44%) between 90 and 179 days. It then fell to 50.72% (95% CI, 34.12–67.32%) between 180 and 209 days and rapidly declined to 42.15% (95% CI, 32.88–51.41%) between 210 and 269 days. Effectiveness continued to decrease to 37.88% (95% CI, 28.88–46.89%) between 270 and 364 days and significantly dropped to 33.23% (95% CI, 23.80–42.66%) between 365 and 639 days (Table 2). Forest plots were presented in the Supplementary Table S11.

**Table 2:** Analysis on the durability of protection against Omicron reinfection with hybrid immunity (incomplete vaccination) (days).^a^

Time from incomplete vaccination to reinfection	Number of articles	Number of studies	Pooled PE estimate and 95% CI	*p*	*I^2^* (%)	*p*	Effect model	*p* for Egger’s test
<60	4	9	66.76% (58.20–75.32%)	<0.001	76.3	<0.001	Random	
60 ~ 89	2	3	64.25% (53.72–74.77%)	<0.001	78.1	0.010	Random	
90 ~ 179	11	35	59.50% (55.56–63.44%)	<0.001	97.8	<0.001	Random	0.159
180 ~ 209	2	7	50.72% (34.12–67.32%)	<0.001	95.1	<0.001	Random	
210 ~ 269	7	13	42.15% (32.88–51.41%)	<0.001	98.8	<0.001	Random	0.656
270 ~ 364	8	12	37.88% (28.88–46.89%)	<0.001	98.1	<0.001	Random	0.022
365 ~ 639	8	15	33.23% (23.80–42.66%)	<0.001	97.8	<0.001	Random	0.839

a The exposed group was hybrid immunity (incomplete vaccination); the control group was never infected and unvaccinated.

### 3.5 Analysis on the durability of protection against Omicron reinfection with hybrid immunity (complete vaccination)

The effectiveness of hybrid immunity (complete vaccination) in mitigating the risk of Omicron variant reinfection showed a diminishing trend over time. This was evident in the observed effectiveness rates of 76.87% (95% CI: 69.43–84.31%) and 76.14% (95% CI: 65.61–86.68%) during days 30–59 and 60–89 post full vaccination, respectively. By days 90–119, the effectiveness decreased to 73.15% (95% CI: 60.78–85.53%) and further declined to 68.68% (95% CI: 60.91–76.45%) by days 120–149. Subsequently, over the intervals of 150–179 days, 180–209 days, and 210–269 days, the effectiveness continued to decline steadily to 64.85% (95% CI, 57.67–72.04%), 63.08% (95% CI, 56.15–70.01%), and 62.97% (95% CI, 60.67–65.26%), respectively. Notably, a significant decrease was observed at 270–364 days, with an effectiveness rate of 54.36% (95% CI, 50.82–57.90%) (Table 3). Forest plots were presented in the Supplementary Table S11.

**Table 3:** Analysis on the durability of protection against Omicron reinfection with hybrid immunity (complete vaccination) (days).^a^

Time from complete vaccination to reinfection	Number of articles	Number of studies	Pooled PE Estimate and 95% CI	*p*	*I^2^* (%)	*p*	Effect model	*p* for Egger’s test
30 ~ 59	5	17	76.87% (69.43–84.31%)	<0.001	99.3	<0.001	Random	0.898
60 ~ 89	2	9	76.14% (65.61–86.68%)	<0.001	99.8	<0.001	Random	
90 ~ 119	2	9	73.15% (60.78–85.53%)	<0.001	99.6	<0.001	Random	
120 ~ 149	3	9	68.68% (60.91–76.45%)	<0.001	90.9	<0.001	Random	
150 ~ 179	1	3	64.85% (57.67–72.04%)	<0.001	0.0	0.700	Fixed	
180 ~ 209	1	4	63.08% (56.15–70.01%)	<0.001	0.00	0.801	Fixed	
210 ~ 269	3	5	62.97% (60.67–65.26%)	<0.001	0.00	0.720	Fixed	
270 ~ 364	1	2	54.36% (50.82–57.90%)	<0.001	0.00	0.438	Fixed	

a The exposed group was hybrid immunity (complete vaccination); the control group was never infected and unvaccinated.

### 3.6 Analysis on the durability of protection against Omicron reinfection with hybrid immunity (booster vaccination)

Hybrid immunity (complete vaccination plus booster dose) demonstrated higher efficacy in mitigating the risk of Omicron reinfection with a more gradual decline over 120 days. The effectiveness of preventing reinfection reached 78.26% (95% CI: 74.87–81.65%) within 30–59 days post-booster vaccination, slightly decreasing to 78.14% (95% CI: 70.91–85.37%) within 60–89 days, and further weakening to 73.49% (95% CI: 68.95–78.04%) within 90–119 days (Table 4). Forest plots were presented in the Supplementary Table S11.

**Table 4:** Analysis on the durability of protection against Omicron reinfection with hybrid immunity (booster vaccination) (days).^a^

Time from booster vaccination to reinfection	Number of articles	Number of studies	Pooled PE Estimate and 95% CI	*p*	*I^2^* (%)	*p*	Effect model	*p* for Egger’s test
30 ~ 59	5	8	78.26% (74.87–81.65%)	<0.001	97.7	<0.001	Random	
60 ~ 89	5	11	78.14% (70.91–85.37%)	<0.001	99.5	<0.001	Random	0.079
90 ~ 119	1	4	73.49% (68.95–78.04%)	<0.001	0.00	0.759	Fixed	

a The exposed group was hybrid immunity (booster vaccination); the control group was never infected and unvaccinated.

### 3.7 Sensitivity analysis and publication bias

**Figure 3:**
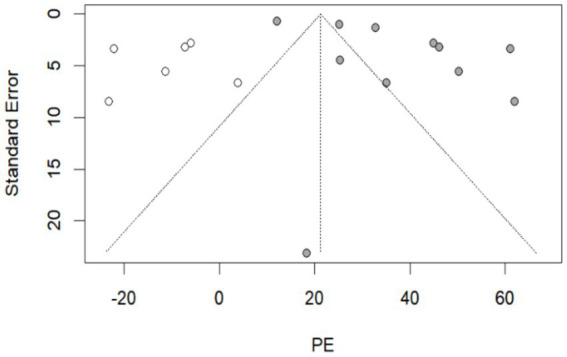
The resulting graph of publication bias was assessed by the trim-and-fill method in the group of 270–364 days after hybrid immunity with incomplete vaccination. Additional funnel plots were presented in the Supplementary Table S12. 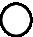: this legend represented supplementary studies. The trim-and-fill method supplemented 6 studies. 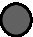: This legend represented the included studies in the group of 270–364 days after hybrid immunity with incomplete vaccination.

By recalculating the pooled estimates after eliminating the studies individually, the new pooled estimates did not change significantly from the original pooled estimates in each group, indicating good stability of the results (Supplementary Table S12). Egger’s test indicated that in the group of 270–364 days after hybrid immunity with incomplete vaccination, *p* = 0.022 (Table 2). Meanwhile, combined with the distribution of the funnel plot, no obvious symmetry was observed (Supplementary Table S12), and publication bias existed in this group. No obvious publication bias was found in the remaining groups (*p* > 0.05). The nonparametric trim-and-fill correction was implemented for the group with publication bias. The Q test showed *p* < 0.001 both before and after trimming. Therefore, the results were analyzed using random-effects models. In the group of 270–364 days after hybrid immunity with incomplete vaccination, the pre-trimming pooled PE = 37.88% (95% CI: 28.88–46.89%, *p* < 0.001), and the post-trimming pooled PE = 21.25% (95% CI: 8.46–34.04, *p* = 0.001). The effect estimates before and after trimming were both statistically significant, which showed that there were no significant changes in the results before and after trimming, suggesting the publication bias had minimal impact and the conclusion was robust (Figure 3).

## 4 Discussion

### 4.1 Hybrid immunity mitigates the risk of reinfection with the Omicron variant to the greatest magnitude

Despite cross-protection between Omicron subvariants, the rapid evolution of the sublineage, along with additional mutation sites and excellent immune escape ability, broke the original immune barrier (4). The results of the present study showed that the hybrid immunity provided greater protection against Omicron reinfection than natural or vaccine-induced immunity alone. This was consistent with the long-term follow-up results of immunological memory specific for SARS-CoV-2 spike proteins, which suggested that hybrid immunity was associated with stronger memory B-cell as well as T-cell antiviral responses, higher neutralizing antibody titers, and lower risk of COVID-19 reinfection (8). We also found that the vaccine dose received was negatively associated with the risk of Omicron reinfection, with the lowest risk of reinfection after booster vaccination among previously infected individuals. The results of serial experimental studies also showed (58, 59) that the S-IgG titer, neutralizing antibody activity against variants, and S-T cell response increased with increasing vaccine doses in previously infected individuals. The booster dose can effectively activate humoral and cellular immune responses, producing higher levels of antibodies than that after the primary vaccination (59, 60). In particular, the frequency of S glycoprotein-specific memory B cells was significantly increased, which is a key determinant of the body’s ability to respond to emerging variants (61). However, the neutralizing activity conferred by previous vaccine series was also limited to the more immune-escaping Omicron sublineages (6), further supporting the importance of regularly updating coronavirus vaccine portfolios and boosting vaccination.

### 4.2 Booster vaccination improves the durability of hybrid immunity, but there are differences in immunity durability

Our study indicated that the protective effect of hybrid immunity decreased with the duration of vaccination, which was consistent with the study by Joshua et al. showing a trend of diminishing vaccine effectiveness in the population observed immediately after three doses of vaccine or hybrid immunity (2/3 doses) within 6 months of vaccination (12), but at a slower rate than that of vaccine-induced and natural immunity alone (9). In addition, our study also found that the protective effectiveness of complete vaccination in past infectees began to moderately decline 3 months after vaccination, reaching a moderate level at 12 months; the effectiveness increased after receiving a booster shot, remaining at a high level within 4 months. Real-world studies have shown that (62) booster vaccinations extend the interval until reinfection for individuals previously infected. Antibody titers after booster vaccinations stabilize at higher baseline levels over time and decay at a slower rate, thereby improving immune durability (60, 63). However, vaccine immunity to the Omicron variant decreases more rapidly than that to other variants (64). Therefore, for individuals with prior infections, receiving a booster dose besides completing the primary vaccination series enhances immune protection. Vaccination promotion policies should improve public awareness and participation in timely COVID-19 vaccination, especially for high-risk populations, to reduce vaccine hesitancy and the burden of reinfection risk.

The durability of hybrid immunity may be further influenced by the host and the type of previously infected variants. Prior clinical trials of mRNA, adenoviral vector, and inactivated vaccines have demonstrated (65) poor cellular immune responses to variants in older adults, particularly in those of advanced age with an underlying disease. It has also been shown that (66) SARS-CoV-2 antibodies are produced slowly in immunodeficient populations, with a transient appearance of antibodies followed by a rapid turnaround. Thus, vaccination strategies should prioritize the booster immunization of vulnerable populations. Moreover, owing to the strong immune evasion of the Omicron variant, the neutralizing capacity of antibodies generated by infection with pre-Omicron variants was less effective in preventing Omicron reinfection (4).

The durability of hybrid immunity may also be related to the type of vaccine administered and an individual’s vaccination schedule. The prior study concluded that the immune protection with heterologous boosters was greater than that with concurrent homologous boosters (67). Therefore, heterologous sequential booster immunization is recommended for vaccine selection. However, administering vaccines too soon after infection significantly reduces the reinfection time, with intervals of no less than 6 months, as recommended in the study by Sánchez-de Prada et al. (62), while 4–12 months in the study by Javier (68). For individuals with prior infections who have completed primary immunization, it may be reasonable to delay subsequent doses for at least 6 months (9).

### 4.3 Future perspectives and limitations

Our meta-analysis searched a wide range of global studies in major databases, and the long time frame of search enabled us to cover the Omicron epidemic, minimizing the omission of information. Meanwhile, the data of the included studies were basically derived from national electronic medical databases, with a large sample size and good representativeness, allowing the analyses to systematically reveal that hybrid immunity mitigated the risk of reinfection and the durability of protection against the Omicron variant, emphasizing the need for booster vaccination. Future research could delve deeper into the minute differences in the hybrid immunity effects produced by diverse types of vaccine combinations to refine vaccination protocols. At the same time, extensive, long-term observational studies should be initiated to more accurately monitor the evolving longevity of hybrid immunity’s protective effects. Additionally, evaluating the influence of individual genetic profiles and pre-existing health conditions on the efficacy of hybrid immunity would be valuable. Studies could also examine the long-term implications of hybrid immunity across different demographic groups, including the older adult and pediatric populations, thereby laying the groundwork for tailored immunization strategies. Furthermore, while approved vaccines provide immune protection against variant strains, they may not fully prevent immune evasion. The continuous evolution of COVID-19 infections means that the long-term efficacy of hybrid immunity is still uncertain. Future research should explore if hybrid immunity offers cross-protection against new COVID-19 variants and similar infectious diseases, potentially broadening its applicability and value. Additionally, studies could investigate the interaction between hybrid immunity and other preventive measures, such as improved hygiene and healthy lifestyles, to achieve a more holistic understanding of its protective benefits. Another aspect deserving attention is comprehending the degree of psychological and behavioral acceptance and compliance with the hybrid immunity strategy, offering valuable insights for policymakers and implementers.

Our analysis has some inherent limitations, which deserve attention. i) Some analyses included a small number of studies, leading to significant heterogeneity; however, the sources of heterogeneity could not be explored because of data limitations, which may have had an impact on the results. ii) Subgroup analyses of vaccine effectiveness could not be performed because of the lack of data on relative vaccination proportions stratified by demographic characteristics or vaccine type. However, the exposure may differ among different populations, limiting the extrapolation of the results. iii) Since the follow-up time units varied across studies (ranging from days, weeks to months), conducting a unified unit analysis posed challenges, making precise time division difficult. iv) Challenges in obtaining consistent population-representative data, stemming from limitations in available information, indicate that our findings may not be fully representative of any specific population group. v) The effect of natural immunity may be underestimated because of the presence of asymptomatic infected individuals, detection of infection by unreported antigens, and failure to analyze the infecting strain type. vi) Due to the continuous emergence and variations of COVID-19 variant strains, it is difficult to keep abreast of their changing tempo during the process of research design and implementation. Additionally, it is challenging to obtain a sufficient number of studies focusing on other variant strains and hybrid immunity, thereby making it impossible to conduct an analysis of other variant strains in a timely and effective manner. Therefore, we may not have been able to accurately evaluate the universal validity of the hybrid immunity strategy when responding to different COVID-19 variant strains. It is also difficult to determine the extent to which the characteristics of Omicron are distinctive and whether hybrid immunity can generate similar or dissimilar effects on other variant strains. Possible bias should be carefully considered when citing and interpreting the results of this study.

## 5 Conclusion

This meta-analysis demonstrated that individuals with hybrid immunity can greatly mitigate the risk of Omicron reinfection, with the lowest risk of reinfection occurring after boosting immunity. Although the protective effect of hybrid immunity waned over time, it was maintained at a moderate level 12 months after hybrid immunity with complete vaccination and at a high level 4 months after hybrid immunity with booster vaccination, suggesting that vaccination remains an important tool for COVID-19 prevention and control.

## Data Availability

The original contributions presented in the study are included in the article/Supplementary material, further inquiries can be directed to the corresponding authors.
