# Supplementary material for: Meta-analysis of hybrid immunity to mitigate the risk of Omicron variant reinfection
Source: Front Public Health. 2024 Aug 26;12:1457266. doi: 10.3389/fpubh.2024.1457266 (PMC11381385; doi:10.3389/fpubh.2024.1457266)
Supplement: Supplementary file 1 [file Data_Sheet_1.PDF]

Supplemental Data Sheet1. Search strategies used for each database.

Database	Search strategies
PubMed	**#1** (SARS-CoV-2[MeSH Terms] OR SARS-CoV-2 Virus[Title/Abstract] OR SARS CoV 2 Virus[Title/Abstract] OR SARS-CoV-2 Viruses[Title/Abstract] OR Virus, SARS-CoV-2[Title/Abstract] OR 2019 Novel Coronavirus[Title/Abstract] OR 2019 Novel Coronaviruses[Title/Abstract] OR Coronavirus, 2019 Novel[Title/Abstract] OR Novel Coronavirus, 2019[Title/Abstract] OR COVID-19 Virus[Title/Abstract] OR COVID 19 Virus[Title/Abstract] OR COVID-19 Viruses[Title/Abstract]OR Virus, COVID-19[Title/Abstract] OR COVID19 Virus[Title/Abstract] OR COVID19 Viruses[Title/Abstract] OR Virus, COVID19[Title/Abstract] OR Viruses, COVID19[Title/Abstract] OR Coronavirus Disease 2019 Virus[Title/Abstract] OR Severe Acute Respiratory Syndrome Coronavirus2[Title/Abstract] OR SARS Coronavirus 2[Title/Abstract] OR Coronavirus 2, SARS[Title/Abstract] OR 2019-nCoV[Title/Abstract] OR COVID-19[MeSH Terms] OR COVID 19[Title/Abstract] OR 2019-nCoV Infection[Title/Abstract] OR 2019 nCoV Infection[Title/Abstract] OR 2019-nCoV Infections[Title/Abstract] OR Infection, 2019-nCoV[Title/Abstract] OR SARS-CoV-2 Infection[Title/Abstract] OR Infection, SARS-CoV-2[Title/Abstract] OR SARS CoV 2 Infection[Title/Abstract] OR SARS-CoV-2 Infections[Title/Abstract] OR 2019 Novel Coronavirus Disease[Title/Abstract] OR 2019 Novel Coronavirus Infection[Title/Abstract] OR COVID-19 Virus Infection[Title/Abstract] OR COVID 19 Virus Infection[Title/Abstract] OR COVID-19 Virus Infections[Title/Abstract] OR Infection, COVID-19 Virus[Title/Abstract] OR Virus Infection, COVID-19[Title/Abstract] OR Coronavirus Disease 2019[Title/Abstract] OR Disease 2019, Coronavirus[Title/Abstract] OR Coronavirus Disease-19[Title/Abstract] OR Coronavirus Disease 19[Title/Abstract] OR Severe Acute Respiratory Syndrome Coronavirus 2 Infection[Title/Abstract] OR COVID-19 Virus Disease[Title/Abstract] OR COVID 19 Virus Disease[Title/Abstract] OR COVID-19 Virus Diseases[Title/Abstract] OR Disease, COVID-19 Virus[Title/Abstract] OR Virus Disease, COVID-19[Title/Abstract] OR SARS Coronavirus 2 Infection[Title/Abstract] OR 2019-nCoV Disease[Title/Abstract] OR 2019 nCoV Disease[Title/Abstract] OR 2019-nCoV Diseases[Title/Abstract] OR Disease, 2019-nCoV[Title/Abstract] OR COVID-19 Pandemic[Title/Abstract] OR (COVID 19 Pandemic[Title/Abstract] OR Pandemic, COVID-19[Title/Abstract] OR COVID-19 Pandemics[Title/Abstract])<br>**#2** (Reinfection[MeSH Terms] OR Reinfections[Title/Abstract] OR Re-infection[Title/Abstract] OR Re-infections[Title/Abstract] OR Recurrent Infection[Title/Abstract] OR Infection, Recurrent[Title/Abstract] OR Recurrent Infections[Title/Abstract] OR Repeat Infection[Title/Abstract])<br>**#3** (Prior infection[Title/Abstract] OR Previous infection[Title/Abstract] OR Past infection[Title/Abstract] OR Primary infection[Title/Abstract] OR Initial infection[Title/Abstract] OR First infection[Title/Abstract] OR Natural immunity[Title/Abstract] OR Infection acquired immunity[Title/Abstract] OR Non-Specific immunity[Title/Abstract] OR Innate immunity[Title/Abstract] OR Seropositivity[Title/Abstract] OR Antigen positive[Title/Abstract] OR Antibody positive[Title/Abstract] OR Naturally acquired

TABLE S2 (Continued)

Database	Search strategies
PubMed	immunity[Title/Abstract] OR Hybrid immunity[Title/Abstract] OR Hybrid protection [Title/Abstract] OR Adaptive immunity[Title/Abstract] OR Acquired immunity[Title/ Abstract] OR Vaccination[Title/Abstract] OR Active immunization[Title/Abstract] OR Unvaccination[Title/Abstract])<br>**#1** AND **#2** AND **#3**
Web of Science	**#1** (TS=(SARS-CoV-2) OR TS=(SARS-CoV-2 Virus) OR TS=(SARS CoV 2 Virus) OR TS=(SARS-CoV-2 Viruses) OR TS=(Virus, SARS-CoV-2) OR TS=(2019 Novel Coronavirus) OR TS=(2019 Novel Coronaviruses) OR TS=(Coronavirus, 2019 Novel) OR TS=(Novel Coronavirus, 2019) OR TS=(COVID-19 Virus) OR TS=(COVID 19 Virus) OR TS=(COVID-19 Viruses) OR TS=(Virus, COVID-19) OR TS=(COVID19 Virus) OR TS=(COVID19 Viruses) OR TS=(Virus, COVID19) OR TS=(Viruses, COVID19) OR TS=(Coronavirus Disease 2019 Virus) OR TS=(Severe Acute Respiratory Syndrome Coronavirus 2) OR TS=(SARS Coronavirus 2) OR TS=(Coronavirus 2, SARS) OR TS=(2019-nCoV) OR TS=(COVID-19) OR TS=(COVID 19) OR TS=(2019-nCoV Infection) OR TS=(2019 nCoV Infection) OR TS=(2019-nCoV Infections) OR TS=(Infection, 2019-nCoV) OR TS=(SARS-CoV-2 Infection) OR TS=(Infection, SARS-CoV-2) OR TS=(SARS CoV 2 Infection) OR TS=(SARS-CoV-2 Infections) OR TS=(2019 Novel Coronavirus Disease) OR TS=(2019 Novel Coronavirus Infection) OR TS=(COVID-19 Virus Infection) OR TS=(COVID 19 Virus Infection) OR TS=(COVID-19 Virus Infections) OR TS=(Infection, COVID-19 Virus) OR TS=(Virus Infection, COVID-19) OR TS=(Coronavirus Disease 2019) OR TS=(Disease 2019, Coronavirus) OR TS=(Coronavirus Disease-19) OR TS=(Coronavirus Disease 19) OR TS=(Severe Acute Respiratory Syndrome Coronavirus 2 Infection) OR TS=(COVID-19 Virus Disease) OR TS=(COVID 19 Virus Disease) OR TS=(COVID-19 Virus Diseases) OR TS=(Disease, COVID-19 Virus) OR TS=(Virus Disease, COVID-19) OR TS=(SARS Coronavirus 2 Infection) OR TS=(2019-nCoV Disease) OR TS=(2019 nCoV Disease) OR TS=(2019-nCoV Diseases) OR TS=(Disease, 2019-nCoV) OR TS=(COVID-19 Pandemic) OR TS=(COVID 19 Pandemic) OR TS=(Pandemic, COVID-19) OR TS=(COVID-19 Pandemics))<br>**#2** (TS=(Reinfection) OR TS=(Reinfections) OR TS=(Re-infection) OR TS=(Re-infections) OR TS=(Recurrent Infection) OR TS=(Infection, Recurrent) OR TS=(Recurrent Infections) OR TS=(Repeat infection))<br>**#3** (TS=(Prior infection) OR TS=(Previous infection) OR TS=(Past infection) OR TS=(Primary infection) OR TS=(Initial infection) OR TS=(First infection) OR TS=(Natural immunity) OR TS=(Infection acquired immunity) OR TS=(Non-Specific Immunity) OR TS=(Innate Immunity) OR TS=(Seropositivity) OR TS=(Antigen positive) OR TS=(Antibody positive) OR TS=(Naturally acquired immunity) OR TS=(Hybrid immunity) OR TS=(Hybrid protection) OR TS=(Adaptive Immunity) OR TS=(Acquired Immunity) OR TS=(Vaccination) OR TS=(Active Immunization) OR TS=(Unvaccination)<br>**#1** AND **#2** AND **#3**

TABLE S2 (Continued)

Database	Search strategies
Embase	**#1** ('sars cov 2'/exp OR 'sars cov 2' OR 'sars-cov-2 virus':ti,ab,kw OR 'sars cov 2 virus':ti,ab,kw OR 'sars-cov-2 viruses':ti,ab,kw OR 'virus, sars-cov-2':ti,ab,kw OR '2019 novel coronavirus':ti,ab,kw OR '2019 novel coronaviruses':ti,ab,kw OR 'coronavirus, 2019 novel':ti,ab,kw OR 'novel coronavirus, 2019':ti,ab,kw OR 'covid-19 virus':ti,ab,kw OR 'covid 19 virus':ti,ab,kw OR 'covid-19 viruses':ti,ab,kw OR 'virus, covid-19':ti,ab,kw OR 'covid19 virus':ti,ab,kw OR 'covid19 viruses':ti,ab,kw OR 'virus, covid19':ti,ab,kw OR 'viruses, covid19':ti,ab,kw OR 'coronavirus disease 2019 virus':ti,ab,kw OR 'severe acute respiratory syndrome coronavirus 2':ti,ab,kw OR 'sars coronavirus 2':ti,ab,kw OR 'coronavirus 2, sars':ti,ab,kw OR '2019 ncov':ti,ab,kw OR 'covid 19':ti,ab,kw OR '2019-ncov infection':ti,ab,kw OR '2019 ncov infection':ti,ab,kw OR '2019-ncov infections':ti,ab,kw OR 'infection, 2019-ncov':ti,ab,kw OR 'sars-cov-2 infection':ti,ab,kw OR 'infection, sars-cov-2':ti,ab,kw OR 'sars cov 2 infection':ti,ab,kw OR 'sars-cov-2 infections':ti,ab,kw OR '2019 novel coronavirus disease':ti,ab,kw OR '2019 novel coronavirus infection':ti,ab,kw OR 'covid-19 virus infection':ti,ab,kw OR 'covid 19 virus infection':ti,ab,kw OR 'covid-19 virus infections':ti,ab,kw OR 'infection, covid-19 virus':ti,ab,kw OR 'virus infection, covid-19':ti,ab,kw OR 'coronavirus disease 2019':ti,ab,kw OR 'disease 2019, coronavirus':ti,ab,kw OR 'coronavirus disease-19':ti,ab,kw OR 'coronavirus disease 19':ti,ab,kw OR 'severe acute respiratory syndrome coronavirus 2 infection':ti,ab,kw OR 'covid-19 virus disease':ti,ab,kw OR 'covid 19 virus disease':ti,ab,kw OR 'covid-19 virus diseases':ti,ab,kw OR 'disease, covid-19 virus':ti,ab,kw OR 'virus disease, covid-19':ti,ab,kw OR 'sars coronavirus 2 infection':ti,ab,kw OR '2019-ncov disease':ti,ab,kw OR '2019 ncov disease':ti,ab,kw OR '2019-ncov diseases':ti,ab,kw OR 'disease, 2019-ncov':ti,ab,kw OR 'covid-19 pandemic':ti,ab,kw OR 'covid 19 pandemic':ti,ab,kw OR 'pandemic, covid-19':ti,ab,kw OR 'covid-19 pandemics':ti,ab,kw)
	**#2** ('reinfection'/exp OR reinfection OR reinfections:ti,ab,kw OR 're infection':ti,ab,kw OR 're infections':ti,ab,kw OR 'recurrent infection':ti,ab,kw OR 'infection, recurrent':ti,ab,kw OR 'recurrent infections':ti,ab,kw OR 'repeat infection':ti,ab,kw)
	**#3** ('prior infection' OR (prior AND ('infection'/exp OR infection)) OR 'previous infection':ti,ab,kw OR 'past infection':ti,ab,kw OR 'primary infection':ti,ab,kw OR 'initial infection':ti,ab,kw OR 'first infection':ti,ab,kw OR 'natural immunity':ti,ab,kw OR 'infection acquired immunity':ti,ab,kw OR 'non-specific immunity':ti,ab,kw OR 'innate immunity':ti,ab,kw OR seropositivity:ti,ab,kw OR 'antigen positive':ti,ab,kw OR 'antibody positive':ti,ab,kw OR 'naturally acquired immunity':ti,ab,kw OR 'hybrid immunity':ti,ab,kw OR 'hybrid protection':ti,ab,kw OR 'adaptive immunity':ti,ab,kw OR 'acquired immunity':ti,ab,kw OR vaccination:ti,ab,kw OR 'active immunization':ti,ab,kw OR unvaccination:ti,ab,kw)
	**#1** AND **#2** AND **#3**
CNKI	**#1** SARS-CoV-2 OR COVID-19 OR 2019 Novel Coronavirus
	**#2** Reinfection OR Repeat infection
	**#3** Previous infection OR Initial infection OR Prior infection OR Natural immunity

TABLE S2 (Continued)

Database	Search strategies
CNKI	OR Vaccination OR Hybrid immunity
	**#1** AND **#2** AND **#3**
Wangfang	**#1** SARS-CoV-2 OR COVID-19 OR 2019 Novel Coronavirus
	**#2** Reinfection OR Repeat infection
	**#3** Previous infection OR Initial infection OR Prior infection OR Natural immunity OR Vaccination OR Hybrid immunity
	**#1** AND **#2** AND **#3**
