# Supplementary material for: Meta-analysis of hybrid immunity to mitigate the risk of Omicron variant reinfection
Source: Front Public Health. 2024 Aug 26;12:1457266. doi: 10.3389/fpubh.2024.1457266 (PMC11381385; doi:10.3389/fpubh.2024.1457266)
Supplement: Supplementary file 6 [file Table_5.DOCX]

AMSTAR criteria and QUOROM score

| Table S5 AMSTAR evaluation checklist and description | | |
| --- | --- | --- |
| Items | Reported? (Yes/No) | Location where item is reported |
| Priori design? | Yes | Review registration and Design (CRD42024539682) (Methods). Supplemental materials:Table S1. |
|  |  |  |
| Duplicate study selection and data extraction? | Yes | Data extraction and quality assessment (Methods). |
|  |  |  |
| Comprehensive search? | Yes | Data sources and searches (Methods). Supplemental materials: Data sheet 1. |
|  |  |  |
| Publication status used as an inclusion criterion? | Yes | Data sources and searches. Eighth point of study inclusion criteria. (Methods). |
|  |  |  |
| Studies list provided? | Yes | Basic characteristics and quality of included studies (Results). References and Figure 1. Supplemental Table S9 and Table S10. |
|  |  |  |
| Characteristics studies provided? | Yes | Basic characteristics and quality of included studies (Results). |
|  |  |  |
| Scientific quality assessed and documented? | Yes | Quality assessment (Methods). Basic characteristics and quality of included studies (Results). Supplemental materials:Table S2, Table S3 and Table S4. |
|  |  |  |
|  |  |  |
| Table 1 AMSTAR evaluation checklist and description (Continued) | | |
| Items | Reported? (Yes/No) | Location where item is reported |
| Scientific quality used conclusions? | Yes | Discussion |
|  |  |  |
| Methods used to combine findings, correct? | Yes | Data analysis (Methods). Results |
|  |  |  |
| Publication bias assessed? | Yes | Data analysis (Methods). Publication bias(Results). Supplemental materials: Table S12 (Funnel plots). |
|  |  |  |
| Conflict of interest stated? | Yes | Conflict of interest and Funding |
