# Supplementary material for: Meta-analysis of hybrid immunity to mitigate the risk of Omicron variant reinfection
Source: Front Public Health. 2024 Aug 26;12:1457266. doi: 10.3389/fpubh.2024.1457266 (PMC11381385; doi:10.3389/fpubh.2024.1457266)
Supplement: Supplementary file 7 [file Table_6.DOCX]

| Table S6. The QUOROM statement checklist | | | | | | | | |
| --- | --- | --- | --- | --- | --- | --- | --- | --- |
| Heading |  | Subheading |  | Descriptor |  | Reported? (Y/N) |  | Page number |
| Title |  |  |  | Identify the report as a meta-analysis [or systematic review] of RCTs |  | Y |  | 1 |
| Abstract |  |  |  | Use a structured format |  | Y |  | 1-2 |
|  |  |  |  |  |  |  |  |  |
|  |  | Objectives |  | Describe the clinical question explicitly |  | N |  |  |
|  |  |  |  |  |  |  |  |  |
|  |  | Data sources |  | The databases (ie, list) and other information sources |  | Y |  | 1 |
|  |  |  |  |  |  |  |  |  |
|  |  | Review methods |  | The selection criteria (ie, population, intervention, outcome, and study design); methods for validity assessment, data abstraction, and study characteristics, and quantitative data synthesis in sufficient detail to permit replication |  | Y |  | 1-2 |
|  |  |  |  |  |  |  |  |  |
|  |  | Results |  | Characteristics of the RCTs included and excluded; qualitative and quantitative findings (ie, point estimates and confidence intervals); and subgroup analyses |  | Y |  | 2 |
|  |  |  |  |  |  |  |  |  |
|  |  | Conclusion |  | The main results |  | Y |  | 2 |
|  |  |  |  |  |  |  |  |  |
|  |  |  |  | Describe |  |  |  |  |
| Introduction |  |  |  | The explicit clinical problem, biological rationale for the intervention, and rationale for review |  | N |  |  |
|  |  |  |  |  |  |  |  |  |
| Table 2 (Continued) |  |  |  |  |  |  |  |  |
| Heading |  | Subheading |  | Descriptor |  | Reported? (Y/N) |  | Page number |
| Methods |  | Searching |  | The information sources, in detail (eg, databases, registers, personal files, expert informants, agencies, hand-searching), and any restrictions (years considered, publication status, language of publication) |  | Y |  | 4 |
|  |  |  |  |  |  |  |  |  |
|  |  | Selection |  | The inclusion and exclusion criteria (defining population, intervention, principal outcomes, and study design |  | Y |  | 4-5 |
|  |  |  |  |  |  |  |  |  |
|  |  | Validity assessment |  | The criteria and process used (eg, masked conditions, quality assessment, and their findings |  | Y |  | 5 |
|  |  |  |  |  |  |  |  |  |
|  |  | Data abstraction |  | The process or processes used (eg, completed independently, in duplicate) |  | Y |  | 5 |
|  |  |  |  |  |  |  |  |  |
|  |  | Study characteristics |  | The type of study design, participants’ characteristics, details of intervention, outcome definitions, &c, and how clinical heterogeneity was assessed |  | Y |  | 4-6 |
|  |  |  |  |  |  |  |  |  |
|  |  | Quantitative data synthesis |  | The principal measures of effect (eg, relative risk), method of combining results (statistical testing and confidence intervals), handling of missing data; how statistical heterogeneity was assessed; a rationale for any a-priori sensitivity and subgroup analyses; and any assessment of publication bias |  | Y |  | 6 |
|  |  |  |  |  |  |  |  |  |
| Table 2 (Continued) |  |  |  |  |  |  |  |  |
| Heading |  | Subheading |  | Descriptor |  | Reported? (Y/N) |  | Page number |
| Results |  | Trial flow |  | Provide a meta-analysis profile summarising trial flow (see figure) |  | Y |  | 23 |
|  |  |  |  |  |  |  |  |  |
|  |  | Study characteristics |  | Present descriptive data for each trial (eg, age, sample size, intervention, dose, duration, follow-up period) |  | Y |  | Supplemental Table S10 |
|  |  |  |  |  |  |  |  |  |
|  |  | Quantitative data synthesis |  | Report agreement on the selection and validity assessment; present simple summary results (for each treatment group in each trial, for each primary outcome); present data needed to calculate effect sizes and confidence intervals in intention-to-treat analyses (eg 2x2 tables of counts, means and SDs, proportions) |  | Y |  | 6-9 |
|  |  |  |  |  |  |  |  |  |
| Discussion |  |  |  | Summarise key findings; discuss clinical inferences based on internal and external validity; interpret the results in light of the totality of available evidence; describe potential biases in the review process (eg, publication bias); and suggest a future research agenda |  | Y |  | 9-12 |
| Quality of reporting of meta-analyses |  |  |  |  |  |  |  |  |
