# Supplementary material for: Meta-analysis of hybrid immunity to mitigate the risk of Omicron variant reinfection
Source: Front Public Health. 2024 Aug 26;12:1457266. doi: 10.3389/fpubh.2024.1457266 (PMC11381385; doi:10.3389/fpubh.2024.1457266)
Supplement: Supplementary file 8 [file Table_7.DOCX]

***Supplementary Material***

**Table 7**

**Note:** The purpose of this search with the inclusion criterion mainly being meta-analysis is to find systematic reviews and meta-analyses that may be related to our study “Meta-analysis of hybrid immunity to mitigate the risk of Omicron variant reinfection”.

**Catalogue**

[Search strategies used for each database. 1](#_Toc173059588)

[Study inclusion and exclusion criteria 2](#_Toc173059589)

[Study selection 2](#_Toc173059590)

[Preferred Reporting Items for Systematic Reviews and Meta-Analyses (PRISMA model) flow map of article selection from the previous literature search. 3](#_Toc173059591)

# Search strategies used for each database.

Search from 1 November 2020 to 14 July 2024.

| TABLE S1. Search strategies used for each database. | |
| --- | --- |
| Database | Search strategies |
| PubMed | **#1**(meta-analysis[Title/Abstract] OR (systematic reviews and meta-regression[Title/Abstract] OR systematic reviews and meta-analysis[Title/Abstract] OR quantitative synthesis[Title/Abstract]  **#2** Omicron[Title/Abstract]  **#3** (Hybrid immunity [Title/Abstract] OR Prior infection[Title/Abstract] OR  Previous infection[Title/Abstract] OR Past infection[Title/Abstract] OR Primary infection[Title/Abstract] OR Natural immunity[Title/Abstract] OR  Vaccination[Title/Abstract] OR vaccine[Title/Abstract] OR Acquired immunity[Title/Abstract] )  **#1** AND **#2** AND **#3** |
|  |  |
| Web  of Science | **#1** ('meta analysis':ti,ab,kw OR 'meta analysis' OR ('systematic reviews':ti,ab,kw AND 'meta regression':ti,ab,kw) OR ('systematic reviews':ti,ab,kw AND 'meta analysis':ti,ab,kw) OR 'quantitative synthesis':ti,ab,kw)  **#2** omicron:ti,ab,kw  **#3** ('hybrid immunity':ti,ab,kw OR 'hybrid immunity' OR (('hybrid'/exp OR hybrid) AND ('immunity'/exp OR immunity)) OR 'prior infection':ti,ab,kw OR 'previous infection':ti,ab,kw OR 'past infection':ti,ab,kw OR 'primary infection':ti,ab,kw OR 'natural immunity':ti,ab,kw OR vaccination:ti,ab,kw OR vaccine:ti,ab,kw OR 'acquired immunity':ti,ab,kw)  **#1** AND **#2** AND **#3** |
|  |  |
| Embase | **#1** (((TS=(meta-analysis)) OR TS=(ystematic reviews and meta-regression)) OR TS=(systematic reviews and meta-analysis)) OR TS=(quantitative synthesis )  **#2** TS=(Omicron)  **#3** ((((((((TS=(Hybrid immunity)) OR TS=(Prior infection)) OR TS=(Previous infection)) OR TS=(Past infection)) OR TS=(Primary infection)) OR TS=(Natural immunity)) OR TS=(Vaccination)) OR TS=(vaccine)) OR TS=(Acquired immunity)  **#1** AND **#2** AND **#3** |
|  |  |

# Study inclusion and exclusion criteria

The inclusion criteria for this study were centered around meta-analyses, with the theme being the impact of hybrid immunity on reinfection with Omicron. The study must provide the necessary statistical data or effect

sizes required for a meta-analysis of effect sizes. There are no language restrictions. To ensure the selection of high-quality studies, strict exclusion criteria were applied.

Exclusion criteria: (1) systematic reviews without data pooling or meta-analysis, narrative reviews, reviews lacking clear selection criteria for included studies and reviews without a specified search algorithm; (2) duplicate publications and literature without original sources; (3) literature unrelated to the current research topic (e.g., antibody, clinical cases with a history of specific diseases, etc); (4) reinfection with non-Omicron variants and unspecified types of reinfection variants; (5) no information provided on included studies; (6) non-observational studies.

# Study selection

Two authors meticulously reviewed and assessed the titles and abstracts of potential studies, diligently identifying relevant manuscript titles, abstracts and full text. Any disagreement was discussed and resolved with the third author.

# Preferred Reporting Items for Systematic Reviews and Meta-Analyses (PRISMA model) flow map of article selection from the previous literature search.

**Identification of studies via databases and registers**

250 articles from databases:

Embase (n=111)

Pubmed (n=61)

Web of Science (n=78)

Wanfang Data

p

**Identification**

Excluding duplicates (n=127)

Exclude articles after reading the titles and abstracts (n=98), for reasons:

(1) Irrelevant to the research topic (e.g., antibody, clinical cases with a history of specific diseases, etc) (n=79)

(2) Systematic reviews, narrative reviews, without data pooling, reviews without a specified search algorithm (n=9)

(3) Unspecified reinfection with omicron variants (n=3)

(4) Reinfection with non-Omicron (5)

(5) No comparison group (2)

Screening of 123 articles

**Screening**

25 full-text articles screened

23 articles excluded, for reasons：

(1) No information provided on included studies (n=1)

(2) lacking clear selection criteria for included studies (n=13)

(3) Non-observational studies (n=4)

(4) Comment (n=2)

(5) Irrelevant to the research topic (n=3)

**Included**

2 articles included in

Meta-analysis

Figure S1. Flow chart of study selection
