# Supplementary material for: Meta-analysis of hybrid immunity to mitigate the risk of Omicron variant reinfection
Source: Front Public Health. 2024 Aug 26;12:1457266. doi: 10.3389/fpubh.2024.1457266 (PMC11381385; doi:10.3389/fpubh.2024.1457266)
Supplement: Supplementary file 10 [file Table_9.DOCX]

**Identification of studies via databases and registers**

Excluding duplicates (n=1452)

4331articles from databases:

Embase (n=1501)

Pubmed (n=652)

Web of Science (n=2243)

CNKI (n=16)

Wangfang Data(n=19)

Wanfang Data

p

**Identification**

Exclude articles after reading the titles and abstracts (n=2831), for reasons:

(1) Irrelevant to the research topic (n=2159)

(2) Case reports, Meta-analyses, Systematic reviews, Animal experiments, Unrelated data extraction, Conference papers, etc (n=653)

(3) Reinfection with non-Omicron strains (n=19)

Screening of 2979 articles

**Screening**

115 articles excluded, for reasons：

(1) Can't find full texts (n=4)

(2) Unrelated data extraction/control group inconsistent/no control set (n=63)

(3) Unspecified type of reinfection strain (n=3)

(4) Reinfection with non-Omicron strains (n=2)

(5) No clear definition of reinfection (n=1)

(6) Irrelevant to the research topic (n=42)

148 full-text articles screened

33 articles included in

Meta-analysis

**Included**

Figure 1. Flow chart of study selection

*Consider, if feasible to do so, reporting the number of records identified from each database or register searched (rather than the total number across all databases/registers).

**If automation tools were used, indicate how many records were excluded by a human and how many were excluded by automation tools.
