# Supplementary material for: Meta-analysis of hybrid immunity to mitigate the risk of Omicron variant reinfection
Source: Front Public Health. 2024 Aug 26;12:1457266. doi: 10.3389/fpubh.2024.1457266 (PMC11381385; doi:10.3389/fpubh.2024.1457266)
Supplement: Supplementary file 11 [file Table_10.DOCX]

Table 10. Basic characteristics and quality of included studies.

| **Study** | **Country** | **Stata** | **Study Population** | | **Type**  **of study** | **Time interval  between infections** | **Omicron subvariants** | **Vaccination status** | **Vaccine type*** | **NOS/**  **AHRQ scores** | |
| --- | --- | --- | --- | --- | --- | --- | --- | --- | --- | --- | --- |
| Celine Y  Tan 2023  (***25***) | Singapore | Asia | ≥18 years old | Cohort | | ≥90d | BA.4/BA.5/  XBB | Unvaccinated;complete/booster vaccination | BNT162b2/mRNA-1273 | | 9 |
|  |  |  |  |  | |  |  |  |  | |  |
| Sara Carazo  2022(***26***) | Canada | North America | ≥12 years old | Case-  control | | ≥90d | Not clearly specified | Unvaccinated;incomplete/complete/  booster vaccination | BNT162b2/mRNA-1273 | | 8 |
|  |  |  |  |  | |  |  |  |  | |  |
| Daniela  Michlmayr  2022(***27***) | Denmark | Europe | ≥2 years old | Cohort | | ≥90d | Not clearly specified | Unvaccinated | Not clearly specified | | 9 |
|  |  |  |  |  | |  |  |  |  | |  |
| Shishi Wu 2022(***28***) | Canada | North America | All ages | Cohort | | ≥90d | Not clearly specified | Unvaccinated;incomplete/complete vaccination | BNT162b2/mRNA-1273/ChAdOx1-S | | 8 |
|  |  |  |  |  | |  |  |  |  | |  |
| Victoria Hall 2022  (***29***) | United Kingdom | Europe | Health care workers aged ≥18 years | Cohort | | ≥90d | Not clearly specified | Unvaccinated;incomplete/complete/  booster vaccination | BNT162b2/ChAdOx1-S | | 8 |

Table S10. (Continued)

| **Study** | **Country** | | **Stata** | **Study Populatin** | | **Type**  **of study** | | **Time interval  between infections** | | **Omicron subvariants** | | **Vaccination status** | | **Vaccine type*** | | **NOS/**  **AHRQ**  **scores** |
| --- | --- | --- | --- | --- | --- | --- | --- | --- | --- | --- | --- | --- | --- | --- | --- | --- |
| Eun Jung Jang 2023  (***30***) | South Korea | | Asia | All ages | | Cohort | | ≥45d | | BA.1/BA.2/  BA.5 | | Unvaccinated;incomplete/complete/  booster vaccination | | Not clearly specified | | 8 |
| Ju Hee Lee 2023(***31***) | South Korea | | Asia | All ages | | Case-control | | ≥45d | | BA.1/BA.2 | | Unvaccinated;incomplete/complete/  booster vaccination | | Not clearly specified | | 8 |
|  |  | |  |  | |  | |  | |  | |  | |  | |  |
| Zhang YY 2023(***32)*** | China | | Asia | All ages | | Cross-  sectional | | ≥30d | | BA.5.2/  BF.7 | | Unvaccinated; incomplete/complete/booster vaccination | | Not clearly specified | | 9 |
|  |  | |  |  | |  | |  | |  | |  | |  | |  |
| Chen XQ 2023(***33***) | China | | Asia | All ages | | Cross-  sectional | | >6M | | BA.5.2 | | Unvaccinated; incomplete/complete/booster vaccination | | Not clearly specified | | 8 |
|  |  | |  |  | |  | |  | |  | |  | |  | |  |
| Stephanie L. S. Penetra 2023(***34)*** | Brazil | | South America | All ages | | Cohort | | ≥90d | | BA.1/BA.2 | | Unvaccinated; complete/booster vaccination | | ChAdOx1-S/CoronaVac/Ad26.COV2.S/ BNT162b2 | | 8 |
|  |  | |  |  | |  | |  | |  | |  | |  | |  |
| Table S10. (Continued) | | | | | | | | | | | | | | | | |
| **Study** | **Country** | | **Stata** | **Study Populatin** | | **Type**  **of study** | | **Time interval  between infections** | | **Omicron subvariants** | | **Vaccination status** | | **Vaccine type*** | | **NOS/**  **AHRQ**  **scores** |
| Chuchu Ye 2023(***35)*** | China | | Asia | Age ≥ 12 months | | Cross-  sectional | | Reinfection is defined as between 1 December and 31 December 2022 | | BA5.2/  BF.7 | | Unvaccinated; incomplete/complete/booster vaccination | | Not clearly specified | | 9 |
|  |  | |  |  | |  | |  | |  | |  | |  | |  |
| Andeweg 2022(***36)*** | Netherlands | | Europe | All ages | | Case-control | | ≥30d | | BA.1/BA.2 | | Unvaccinated; complete/booster vaccination | | BNT162b2/mRNA-1273/ChAdOx1-S/ Ad26.COV2.S | | 9 |
|  |  | |  |  | |  | |  | |  | |  | |  | |  |
| Ian D. Plumb 2022(***37)*** | USA | | North America | ≥18 years old | | Case-control | | ≥90d | | Not clearly specified | | Unvaccinated; incomplete/complete/booster vaccination | | BNT162b2/mRNA-1273 | | 8 |
|  |  | |  |  | |  | |  | |  | |  | |  | |  |
| Table S10. (Continued) | | | | | | | | | | | | | | | | |
| **Study** | **Country** | **Stata** | | | **Study Populatin** | | **Type**  **of study** | | **Time interval  between infections** | | **Omicron subvariants** | | **Vaccination status** | | **Vaccine type*** | **NOS/**  **AHRQ**  **scores** |
| Elizabeth T 2022(***38)*** | USA | North America | | | Prison residents aged ≥18 years | | Cohort | | ≥90d | | Not clearly specified | | Unvaccinated; complete/booster vaccination | | mRNA-1273/BNT162b2 | 8 |
|  |  | |  |  | |  | |  | |  | |  | |  | |  |
| Fotinie Ntziora 2022(***39)*** | Greece | | Europe | Health care workers aged ≥18 years | | Cohort | | ≥90d | | BA.1/  BA.2 | | Complete/booster vaccination | | BNT162b2/mRNA-1273 | | 6 |
|  |  | |  |  | |  | |  | |  | |  | |  | |  |
| Annabel A Powell 2023(***40)*** | England | | Europe | 12-17 years old | | Case-  control | | ≥90d | | BA.1/  BA.2 | | Complete vaccination | | BNT162b2/mRNA-1273 | | 7 |
|  |  | |  |  | |  | |  | |  | |  | |  | |  |
|  |  | |  |  | |  | |  | |  | |  | |  | |  |
| Table S10. (Continued) | | | | | | | | | | | | | | | | |
| **Study** | **Country** | | **Stata** | **Study Populatin** | | **Type**  **of study** | | **Time interval  between infections** | | **Omicron subvariants** | | **Vaccination status** | | **Vaccine type*** | | **NOS/**  **AHRQ**  **scores** |
| Katrine Finderup Nielsen 2021(***41)*** | Denmark | | Europe | All ages | | Cohort | | ≥90d | | Not clearly specified | | Unvaccinated;  complete vaccination | | BNT162b2/mRNA-1273/ChAdOx1-S/  Ad26.COV2.S | | 9 |
|  |  | |  |  | |  | |  | |  | |  | |  | |  |
| Jianpeng Cai 2023(***42)*** | China | | Asia | ≥18 years old | | Cross-  sectional | | $\bar{X}$=  257.9 (94-611)d | | BA.5 | | Unvaccinated; incomplete/complete/  booster vaccination | | Not clearly specified | | 9 |
|  |  | |  |  | |  | |  | |  | |  | |  | |  |
| Weien Yu 2023(***43)*** | China | | Asia | Patients with initial infections in three hospitals in Shanghai. Age unknown | | Cohort | | ≥90d | | BA.5.2/  BF.7 | | Incomplete/complete/booster vaccination | | Not clearly specified | | 8 |
|  |  | |  |  | |  | |  | |  | |  | |  | |  |
| Table S10. (Continued) | | | | | | | | | | | | | | | |  |
| **Study** | **Country** | | **Stata** | **Study Populatin** | | **Type**  **of study** | | **Time interval  between infections** | | **Omicron subvariants** | | **Vaccination status** | | **Vaccine type*** | | **NOS/**  **AHRQ**  **scores** |
| Chu YR 2023(***44)*** | China | | Asia | All ages | | Cross-  sectional | | ≥90d | | Not clearly specified | | Unvaccinated; incomplete/complete/booster vaccination | | Not clearly specified | | 8 |
|  |  | |  |  | |  | |  | |  | |  | |  | |  |
| Dani Cohen 2023(***45)*** | Israel | | Europe | ≥18 years old | | Cohort | | ≥90d | | BA.1/BA.2 | | Unvaccinated; incomplete/complete/booster vaccination | | BNT162b2 | | 8 |
| Šmíd 2022(***46)*** | Czechia | | Europe | All ages | | Case-control | | 121-day periods for the time from the last infection | | Not clearly specified | | Unvaccinated; complete/booster vaccination | | BNT162b2/mRNA-1273/ChAdOx1-S/  Ad26.COV2.S | | 8 |
|  |  | |  |  | |  | |  | |  | |  | |  | |  |
| Chin 2022(***47)*** | USA | | North America | ≥18 years old | | Case-control | | ≥90d | | Not clearly specified | | Unvaccinated; booster vaccination | | BNT162b2/mRNA-1273 | | 9 |
|  |  | |  |  | |  | |  | |  | |  | |  | |  |
|  |  | |  |  | |  | |  | |  | |  | |  | |  |
| Table S10. (Continued) | | | | | | | | | | | | | | | | |
| **Study** | **Country** | | **Stata** | **Study Populatin** | | **Type**  **of study** | | **Time interval  between infections** | | **Omicron subvariants** | | **Vaccination status** | | **Vaccine type*** | | **NOS/**  **AHRQ**  **scores** |
| Cerqueira 2022(***48)*** | Brazil | | South America | ≥18 years old | | Case-control | | ≥90d | | Not clearly specified | | Unvaccinated; complete/booster vaccination | | BNT162b2/ChAdOx1-S/Ad26.COV2.S/  CoronaVac | | 9 |
|  |  | |  |  | |  | |  | |  | |  | |  | |  |
| Carazo 2022(***49)*** | Canada | | North America | ≥18 years old | | Case-control | | ≥90d | | Not clearly specified | | Unvaccinated; complete/booster vaccination | | BNT162b2/mRNA-1273 | | 8 |
|  |  | |  |  | |  | |  | |  | |  | |  | |  |
| Altarawneh 2022(***50)*** | Qatar | | Asia | All ages | | Case-control | | ≥90d | | BA.1/BA.2 | | Unvaccinated; complete/booster vaccination | | BNT162b2/mRNA-1273 | | 9 |
| Sara Carazo 2023(***51)*** | Canada | | North America | Health care workers aged≥18 years | | Case-control | | ≥30d | | BA.2 | | Unvaccinated; complete/booster vaccination | | BNT162b2/mRNA-1273 | | 9 |
|  |  | |  |  | |  | |  | |  | |  | |  | |  |
| Christian Holm Hansen 2022(***52)*** | Denmark | | Europe | ≥18 years old | | Case-control | | ≥90d | | BA.5/BA.2 | | Unvaccinated; booster vaccination | | BNT162b2/mRNA-1273 | | 9 |
|  |  | |  |  | |  | |  | |  | |  | |  | |  |
|  |  | |  |  | |  | |  | |  | |  | |  | |  |
| Table S10. (Continued) | | | | | | | | | | | | | | | | |
| **Study** | **Country** | | **Stata** | **Study Populatin** | | **Type**  **of study** | | **Time interval  between infections** | | **Omicron subvariants** | | **Vaccination status** | | **Vaccine type*** | | **NOS/**  **AHRQ**  **scores** |
| Dai  YX 2023(***53)*** | China | | Asia | All ages | | Cross-  sectional | | ≥60d | | BA.5.2 | | Unvaccinated; incomplete/complete/  booster vaccination | | Not clearly specified | | 6 |
|  |  | |  |  | |  | |  | |  | |  | |  | |  |
| Lind 2022(***54)*** | USA | | North America | ≥5 years old | | Case-control | | ≥90d | | BA.1 | | Unvaccinated; booster vaccination | | BNT162b2/mRNA-1273 | | 9 |
| Alena Chalupka 2024(***55)*** | Austria | | Oceania | All ages | | Cohort | | ≥90d | | BA.1/BA.5 | | Unvaccinated; booster vaccination | | BNT162b2 | | 8 |
|  |  | |  |  | |  | |  | |  | |  | |  | |  |
| Håkon Bøås 2024(***56)*** | Norway | | Europe | All ages | | Cohort | | ≥60d | | Not clearly specified | | Complete/booster vaccination | | BNT162b2/mRNA-1273 | | 9 |
|  |  | |  |  | |  | |  | |  | |  | |  | |  |
| Hiam Chemaitelly 2024(***57)*** | Qatar | | Asia | All ages | | Case-control | | ≥90d | | JN.1 | | Unvaccinated | | Not clearly specified | | 8 |

*****The vaccine platforms for the specific vaccine types mentioned in Table 10 include mRNA (BNT162b2, mRNA-1273), adenoviral vector (ChAdOx1-S, Ad26.COV2.S), and inactivated vaccine (CoronaVac).
