# Supplementary material for: Meta-analysis of hybrid immunity to mitigate the risk of Omicron variant reinfection
Source: Front Public Health. 2024 Aug 26;12:1457266. doi: 10.3389/fpubh.2024.1457266 (PMC11381385; doi:10.3389/fpubh.2024.1457266)
Supplement: Supplementary file 12 [file Table_11.DOCX]

***Supplemental Material***

**Table 11**

**Catalog**

**[Forest plots](#_Toc1721)** [1](#_Toc1721)

[Risk analysis of hybrid immunity against reinfection with Omicron variant (Inside the article in Table 1) 1](#_Toc29332)

**[Forest plots](#_Toc6257)** [5](#_Toc6257)

[Analysis on the durability of protection against Omicron reinfection with hybrid immunity (incomplete vaccination) (Inside the article in Table 2) 5](#_Toc16193)

**[Forest plots](#_Toc17289)** [9](#_Toc17289)

[Analysis on the durability of protection against Omicron reinfection with hybrid immunity (complete vaccination) (Inside the article in Table 3) 9](#_Toc18103)

**[Forest plots](#_Toc24203)** [12](#_Toc24203)

[Analysis on the duration of protection against Omicron reinfection with hybrid immunity (booster vaccination) (Inside the article in Table 4) 12](#_Toc32419)

# Forest plots

## Risk analysis of hybrid immunity against reinfection with Omicron variant (Inside the article in Table 1)

**
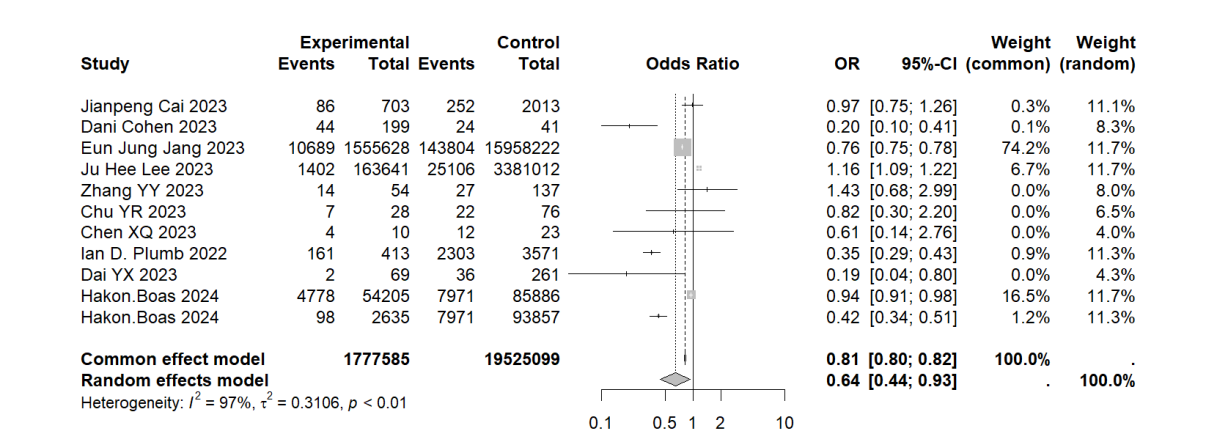
**

FIGURE A. The exposure group is hybrid immunity (incomplete vaccination) and the control group is natural immunity.


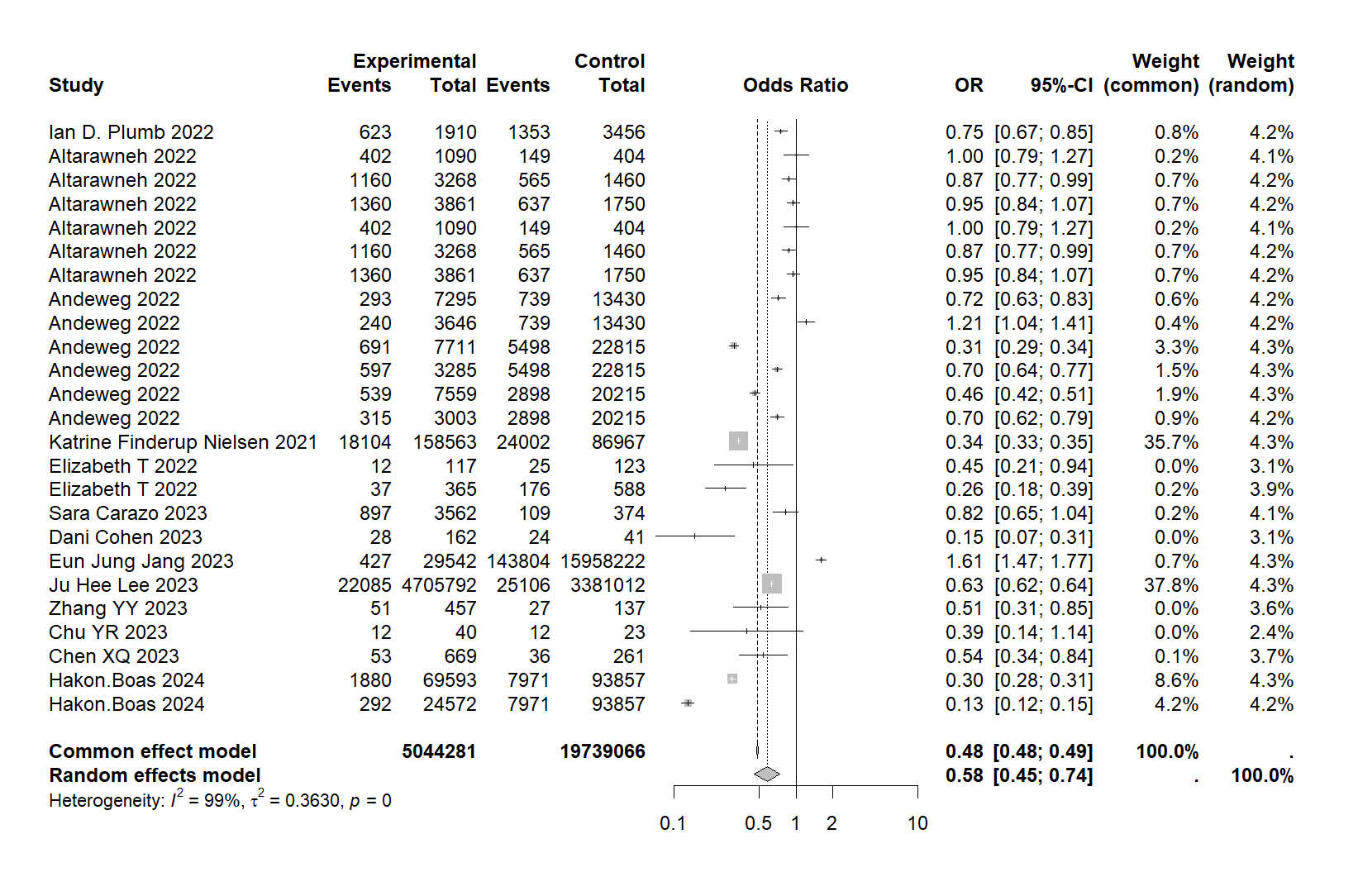


FIGURE B. The exposure group is hybrid immunity (complete vaccination) and the control group is natural immunity.


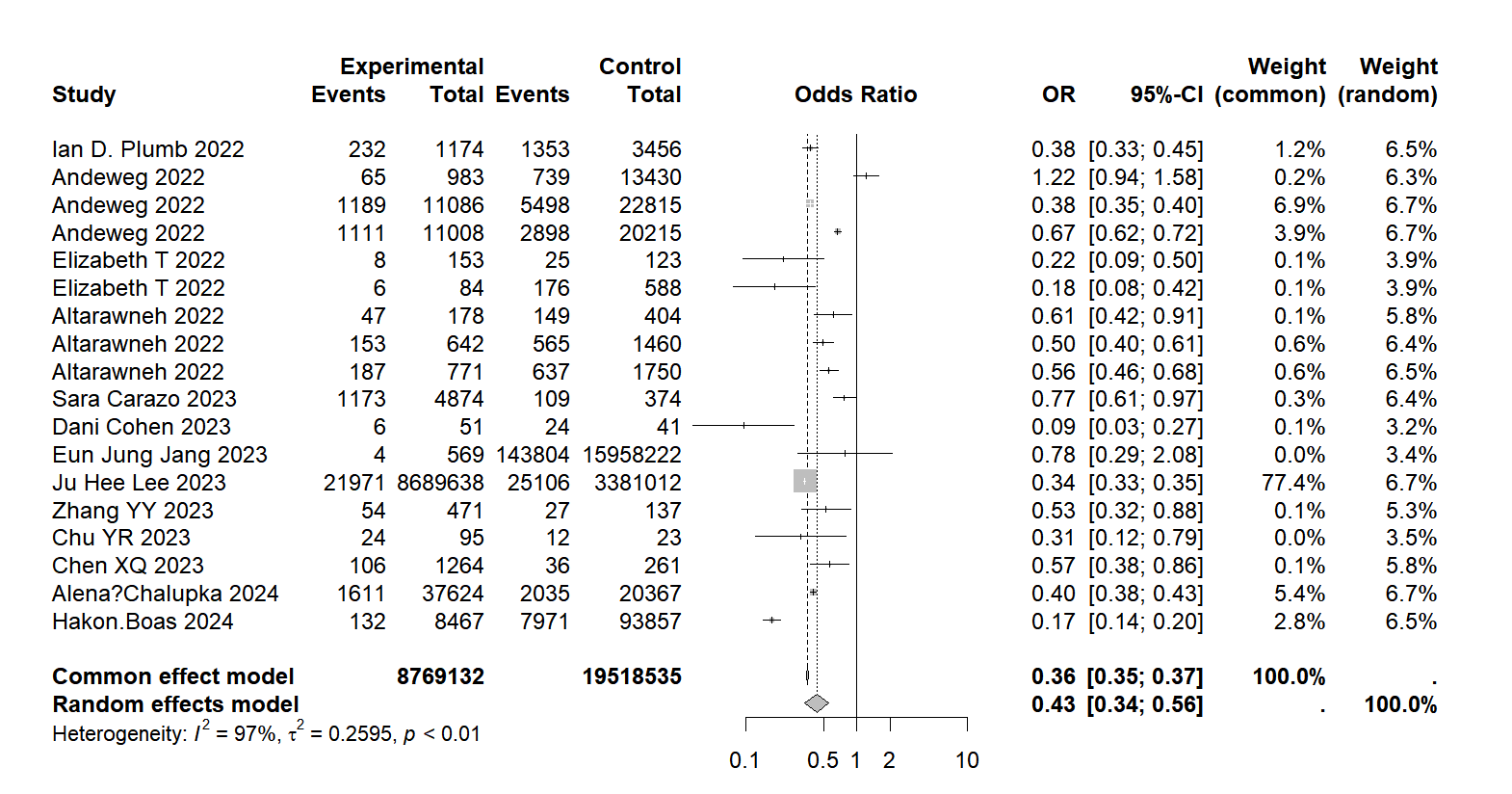


FIGURE C. The exposure group is hybrid immunity (booster vaccination) and the control group is natural immunity.


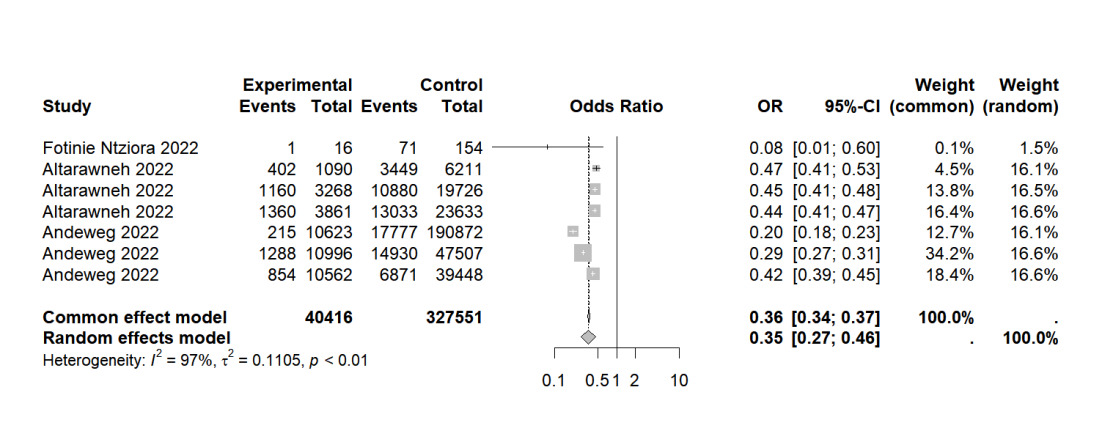


FIGURE D. The exposure group is hybrid immunity (complete vaccination) and the control group is complete vaccination.


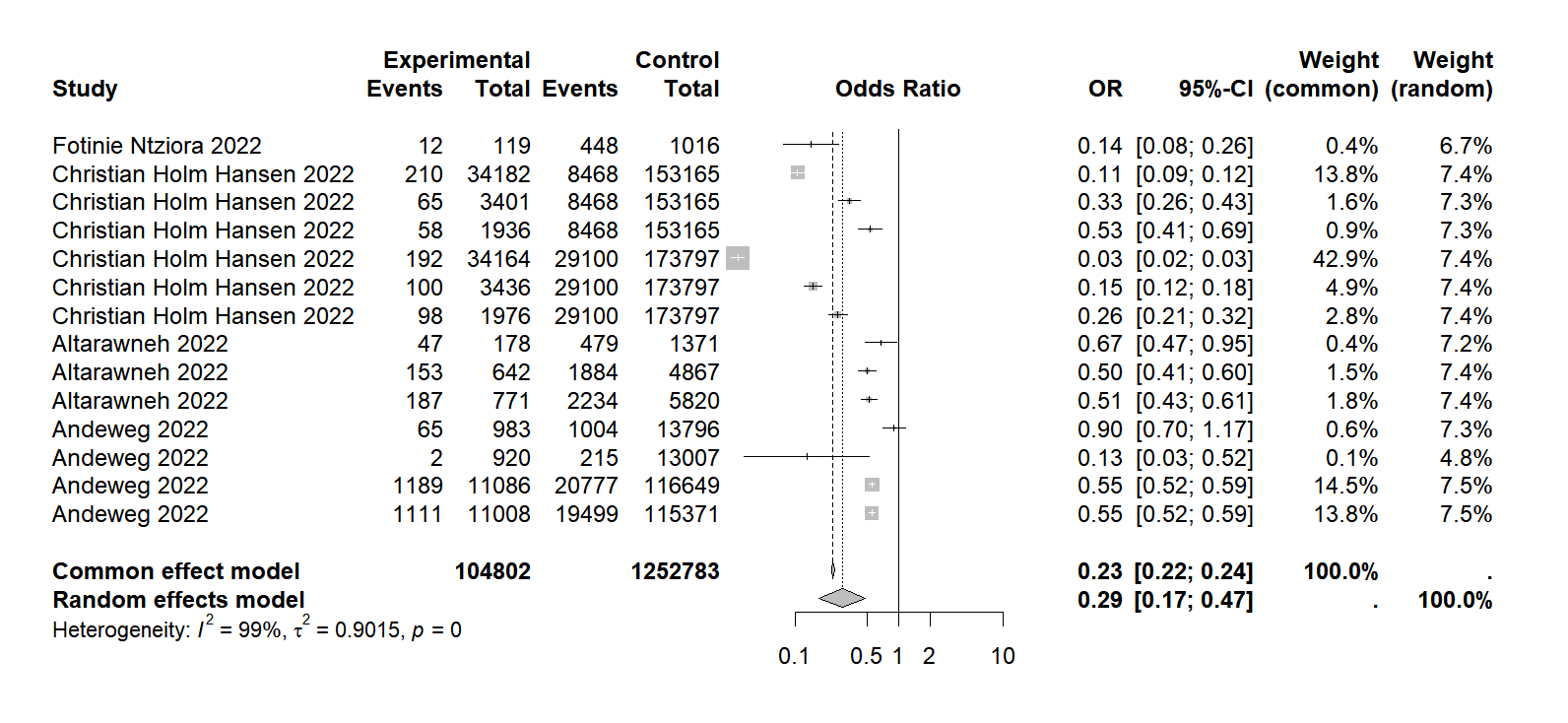


FIGURE E. The exposure group is hybrid immunity (booster vaccination) and the control group is booster vaccination.


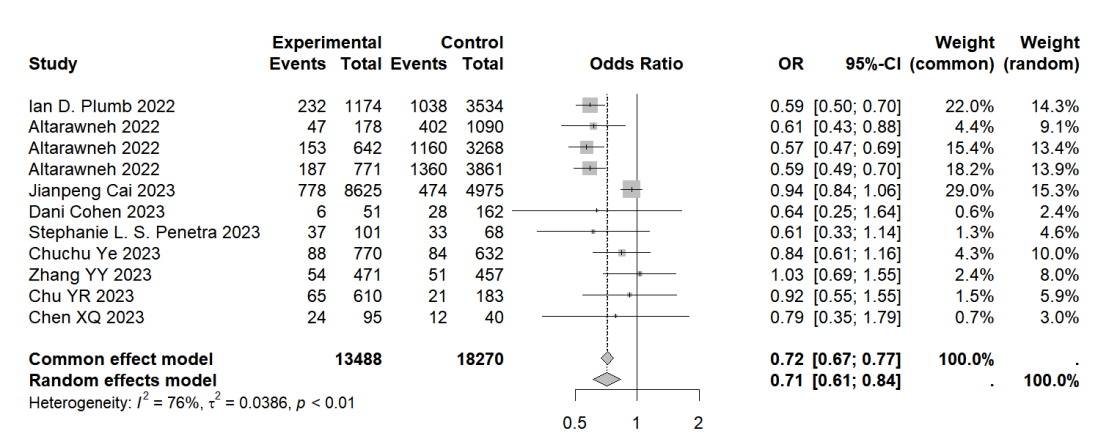


FIGURE F. The exposure group is hybrid immunity (booster vaccination) and the control group is hybrid immunity (complete vaccination).


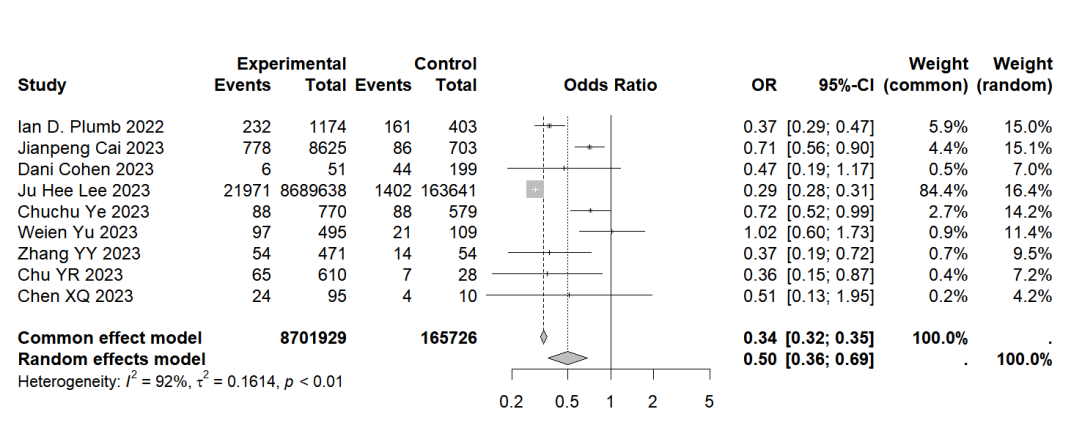


FIGURE G. The exposure group is hybrid immunity (booster vaccination) and the control group is hybrid immunity (incomplete vaccination).


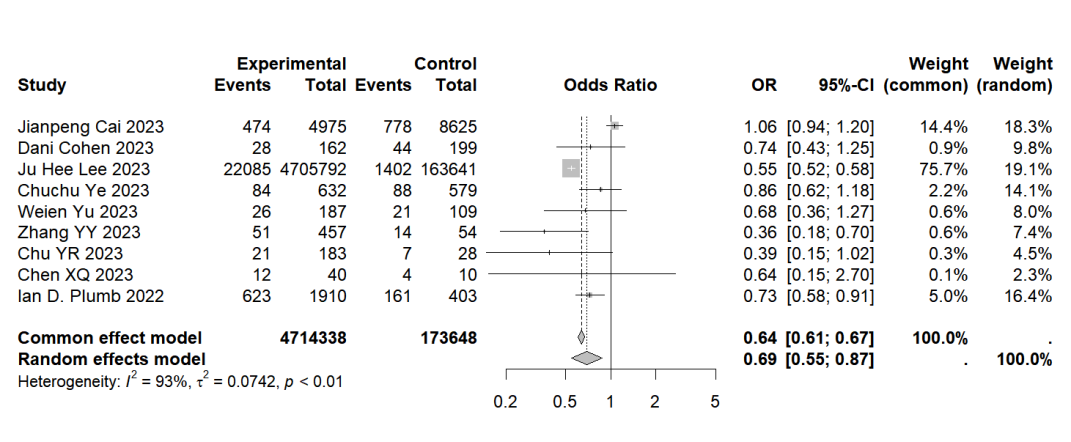


FIGURE H. The exposure group is hybrid immunity (complete vaccination) and the control group is hybrid immunity (incomplete vaccination).

# **Forest plots**

## **Analysis on the durability of protection against Omicron reinfection with hybrid immunity (incomplete vaccination) (Inside the article in** **Table 2)**

**
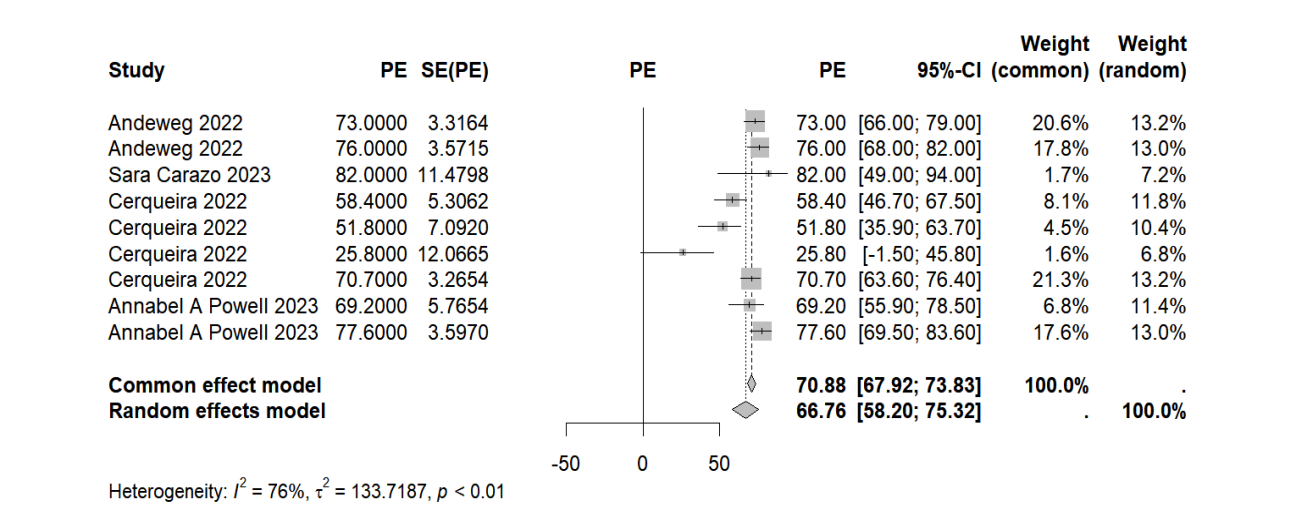
**

FIGURE A. Effectiveness in mitigating Omicron reinfection less than 60 days from hybrid immunity (incomplete vaccination).


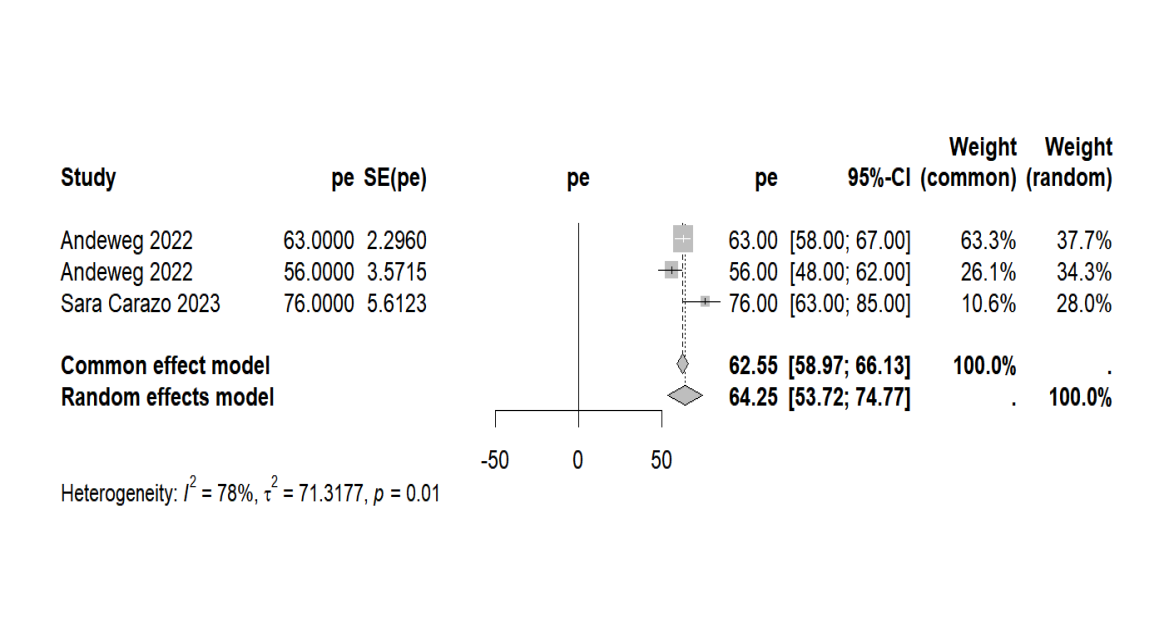


FIGURE B. Effectiveness in mitigating Omicron reinfection after 60-89 days from hybrid immunity (incomplete vaccination).


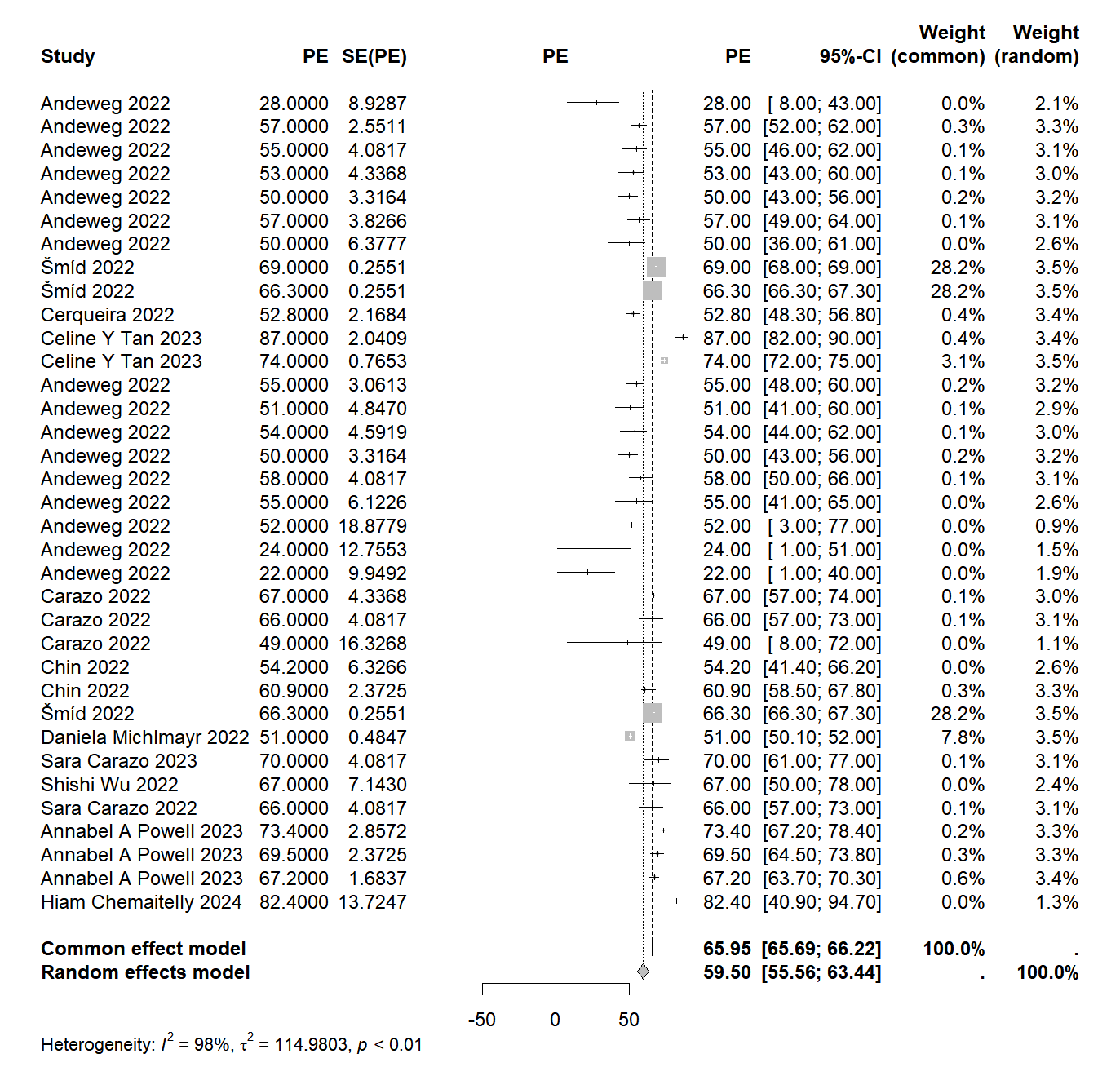


FIGURE C. Effectiveness in mitigating Omicron reinfection after 90-179 days from hybrid immunity (incomplete vaccination).


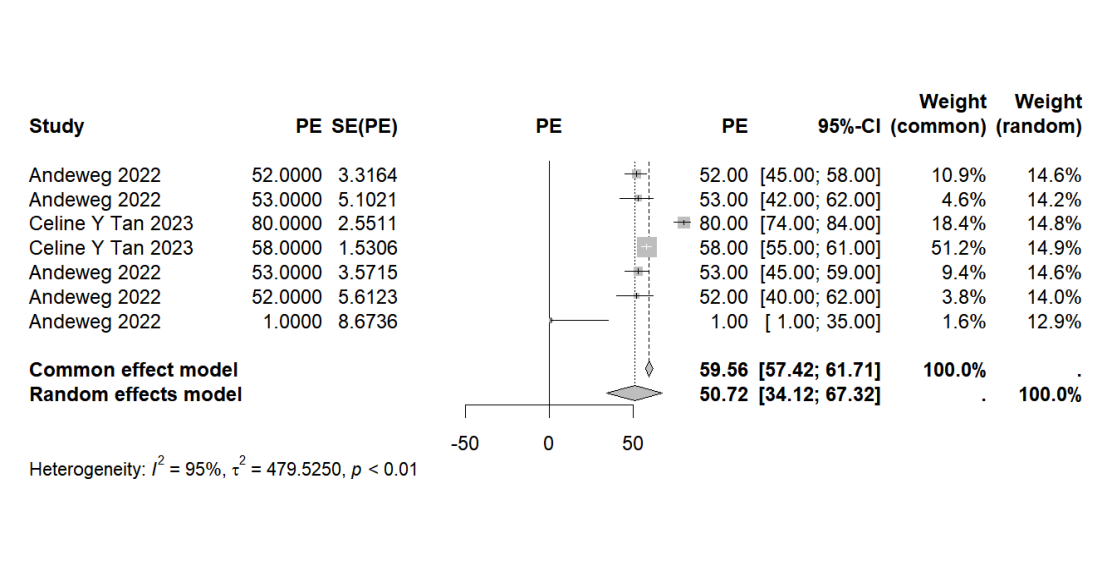


FIGURE D. Effectiveness in mitigating Omicron reinfection after 180-209 days from hybrid immunity (incomplete vaccination).


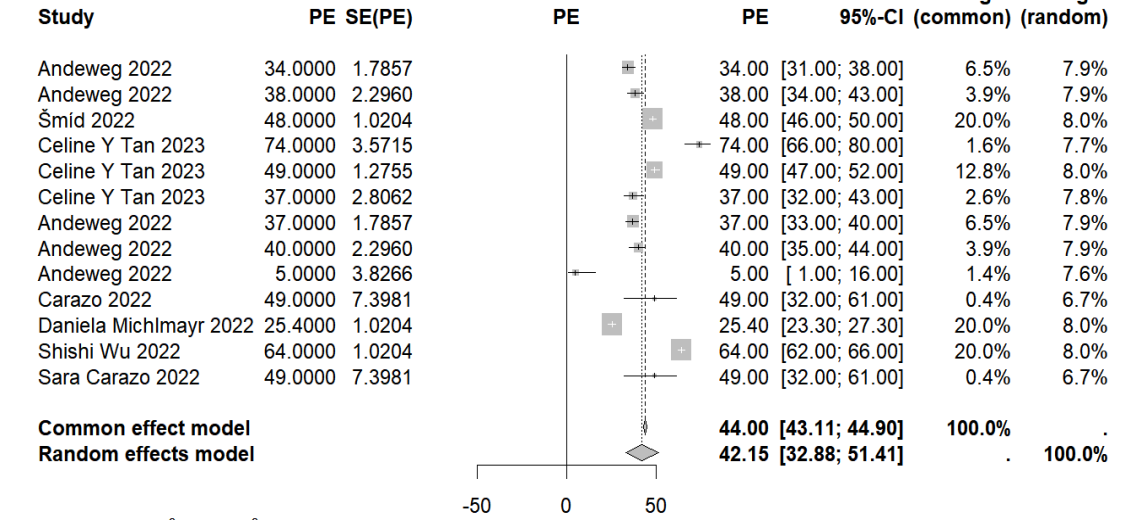


FIGURE E. Effectiveness in mitigating Omicron reinfection after 210-269 days from hybrid immunity (incomplete vaccination).


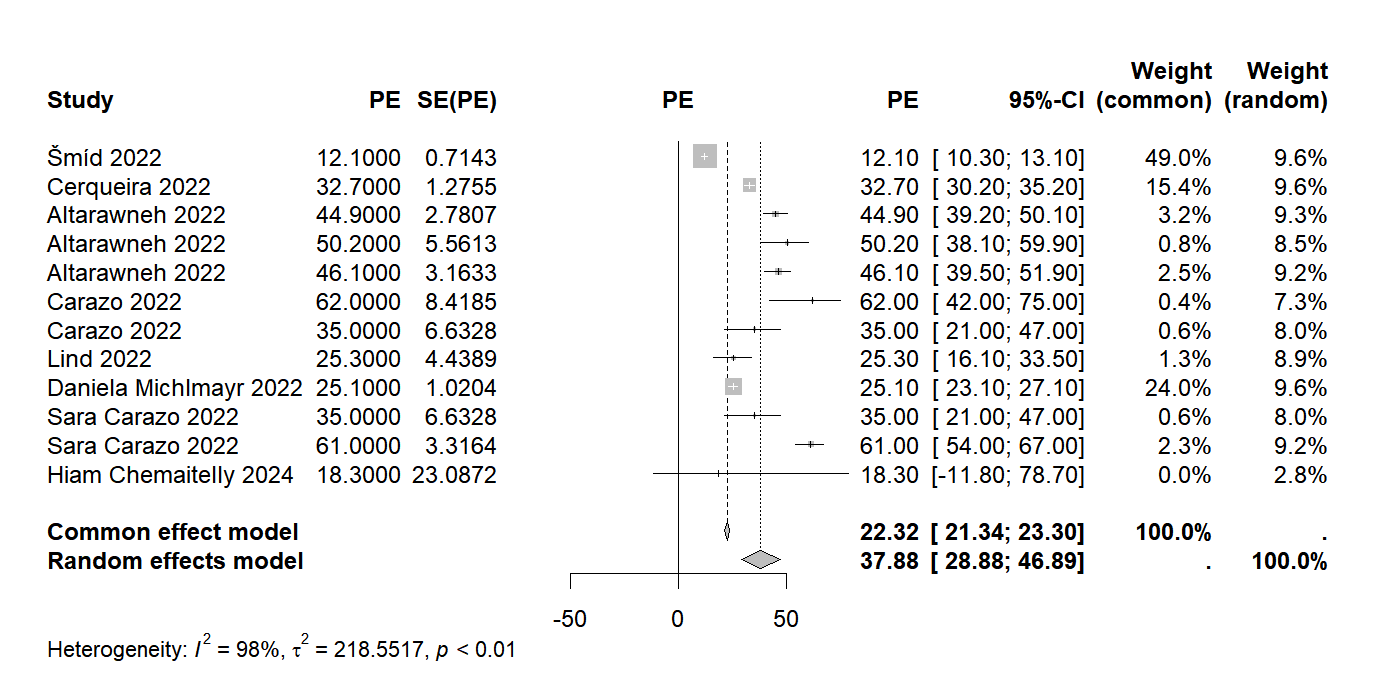


FIGURE F. Effectiveness in mitigating Omicron reinfection after 270-364 days from hybrid immunity (incomplete vaccination).


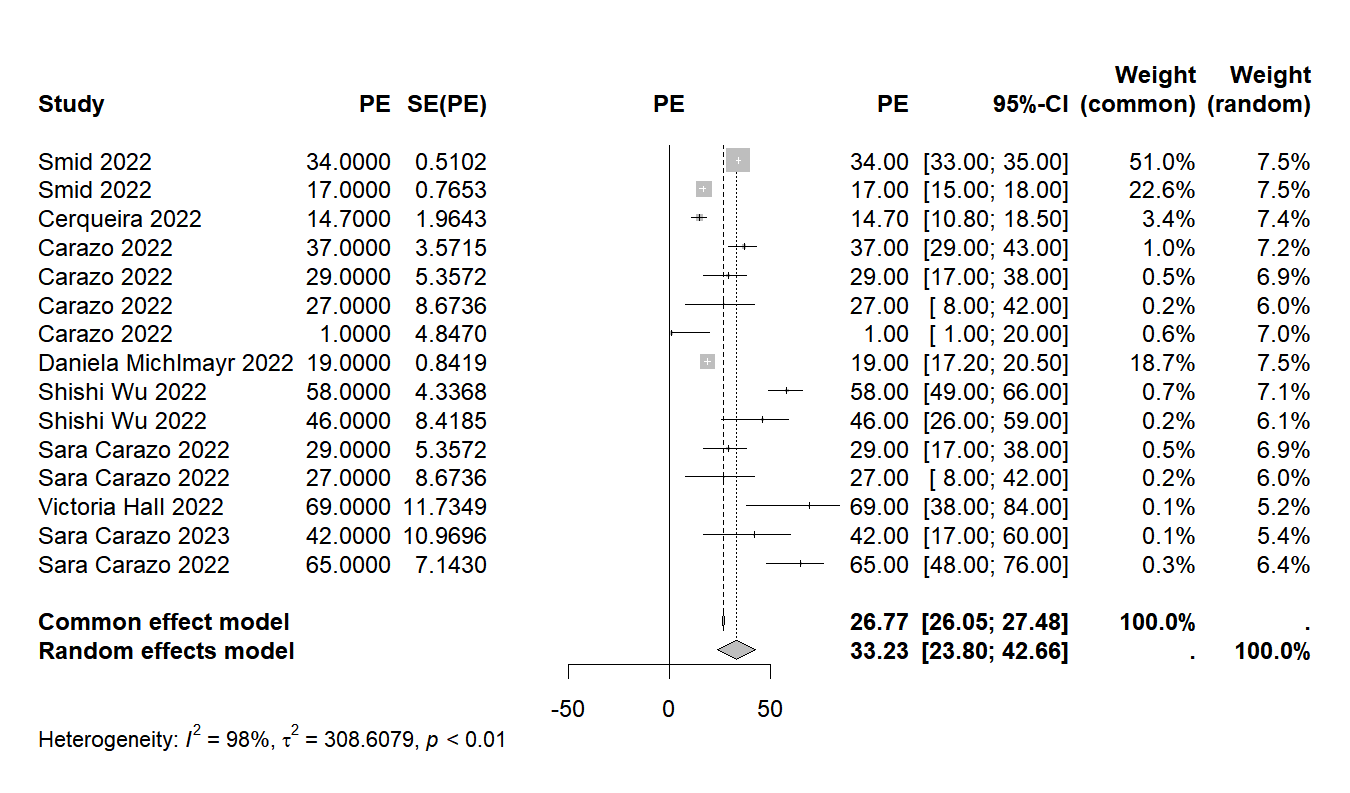


FIGURE G. Effectiveness in mitigating Omicron reinfection after 365-639 days from hybrid immunity (incomplete vaccination).

#

# **Forest plots**

## **Analysis on the durability of protection against Omicron reinfection with hybrid immunity (complete vaccination) (Inside the article in Table 3)**


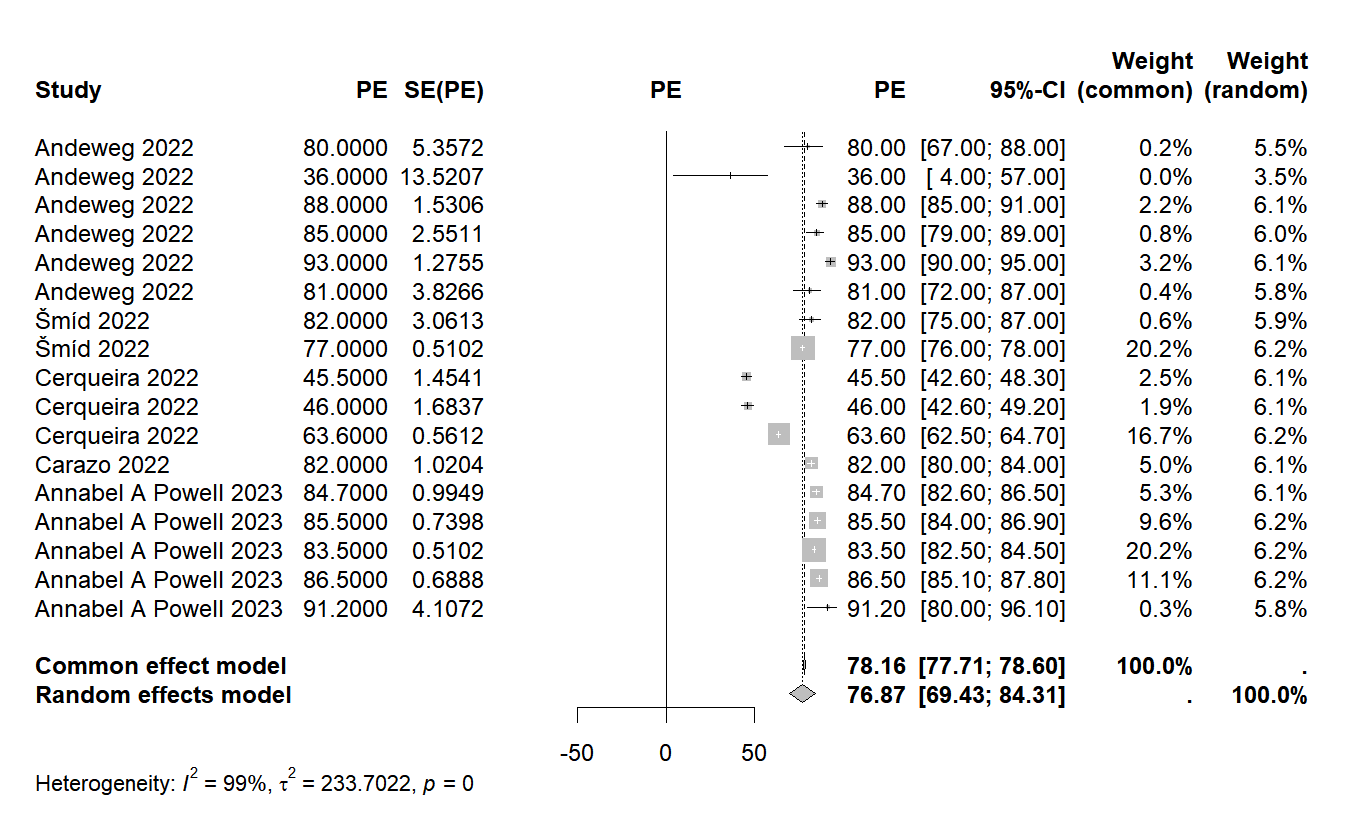


FIGURE A. Effectiveness in mitigating Omicron reinfection after 30-59 days from hybrid immunity (complete vaccination).


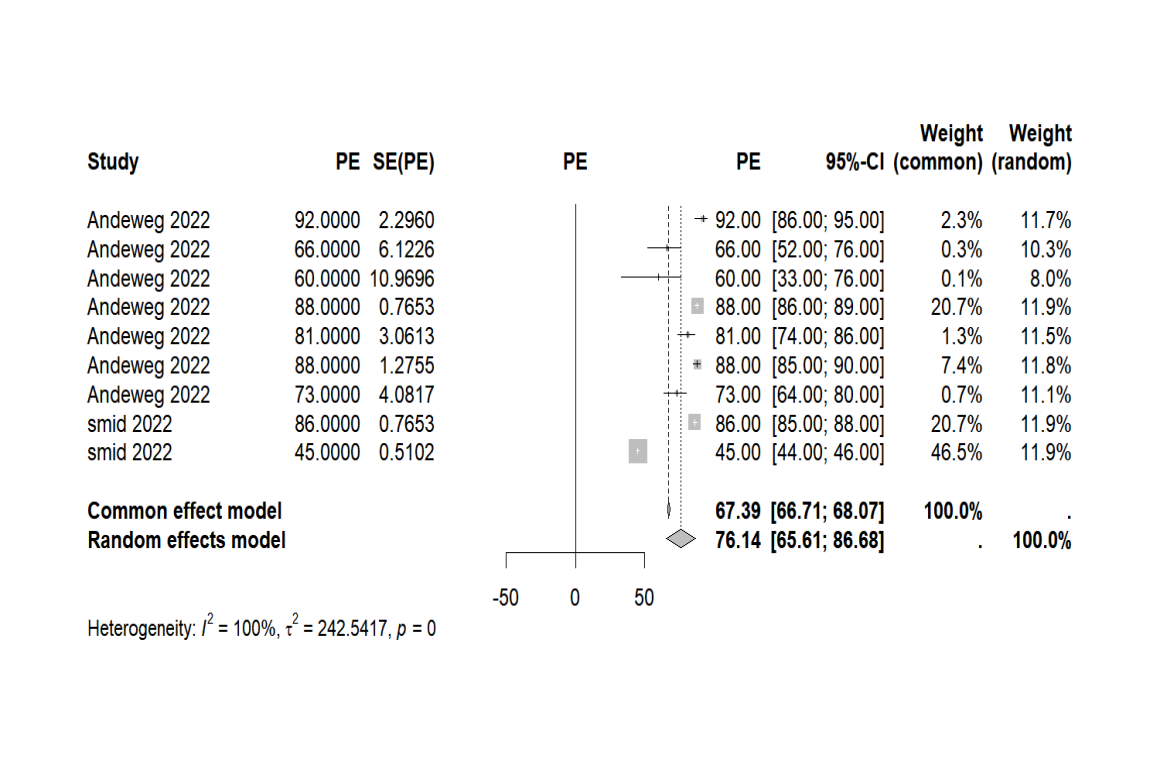


FIGURE B. Effectiveness in mitigating Omicron reinfection after 60-89 days from hybrid immunity (complete vaccination).


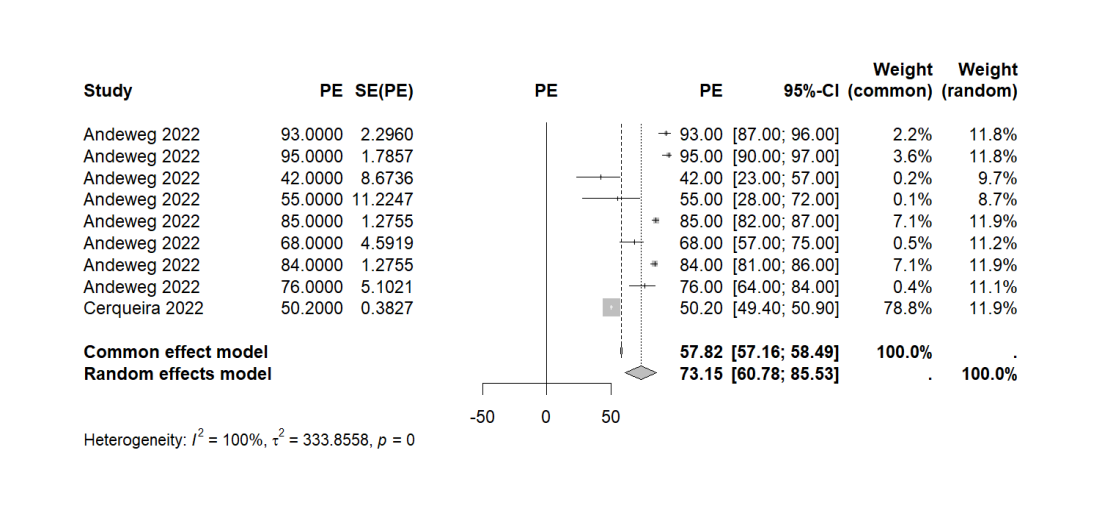


FIGURE C. Effectiveness in mitigating Omicron reinfection after 90-119 days from hybrid immunity (complete vaccination).


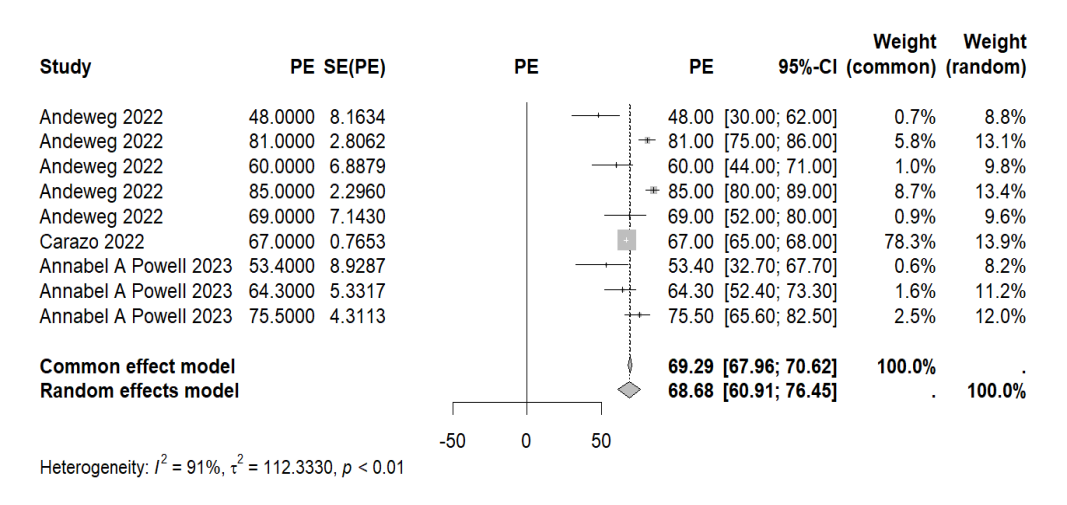


FIGURE D. Effectiveness in mitigating Omicron reinfection after 120-149 days from hybrid immunity (complete vaccination).


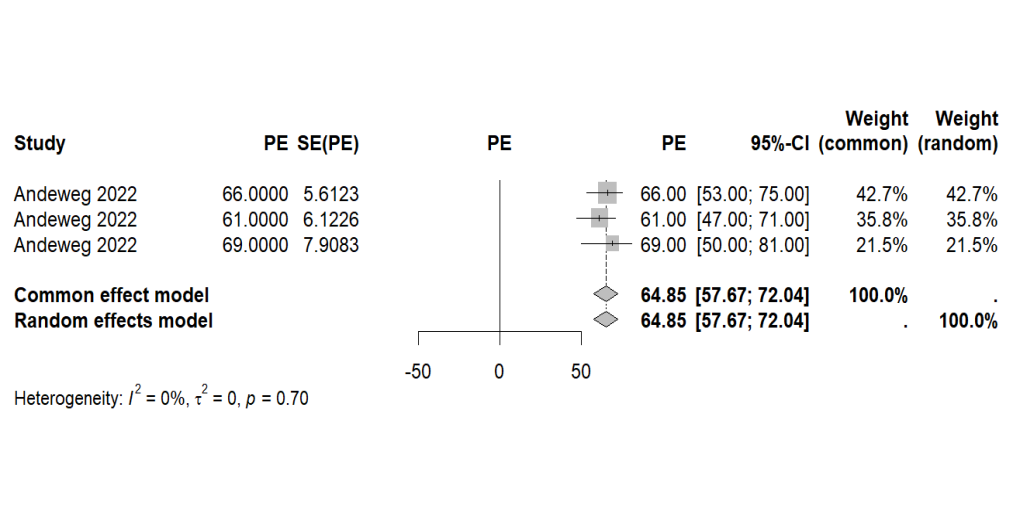


FIGURE E. Effectiveness in mitigating Omicron reinfection after 150-179 days from hybrid immunity (complete vaccination).


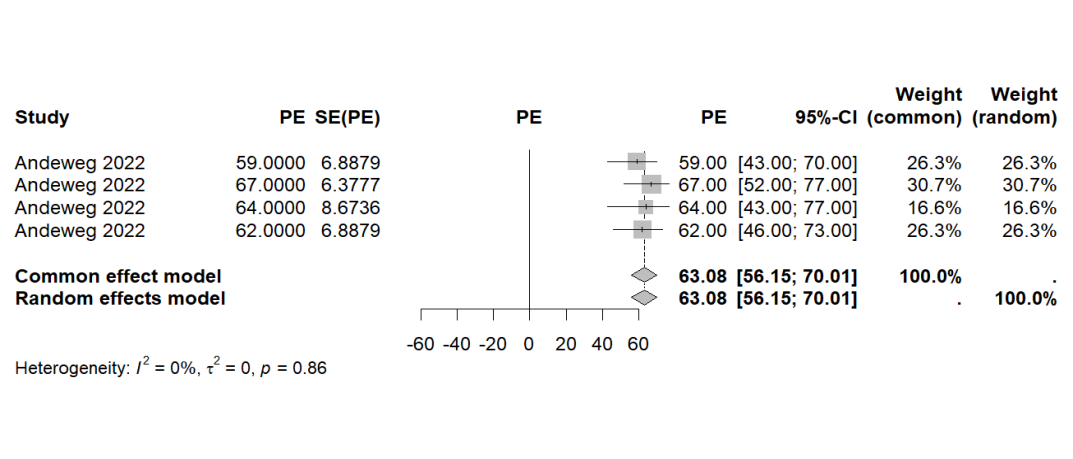


FIGURE F. Effectiveness in mitigating Omicron reinfection after 180-209 days from hybrid immunity (complete vaccination).


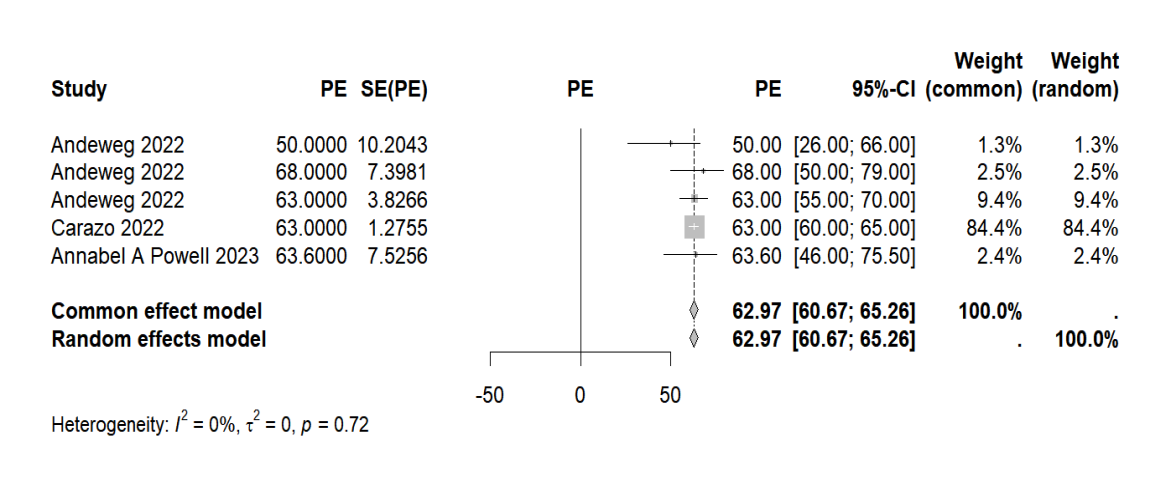


FIGURE G. Effectiveness in mitigating Omicron reinfection after 210-269 days from hybrid immunity (complete vaccination).


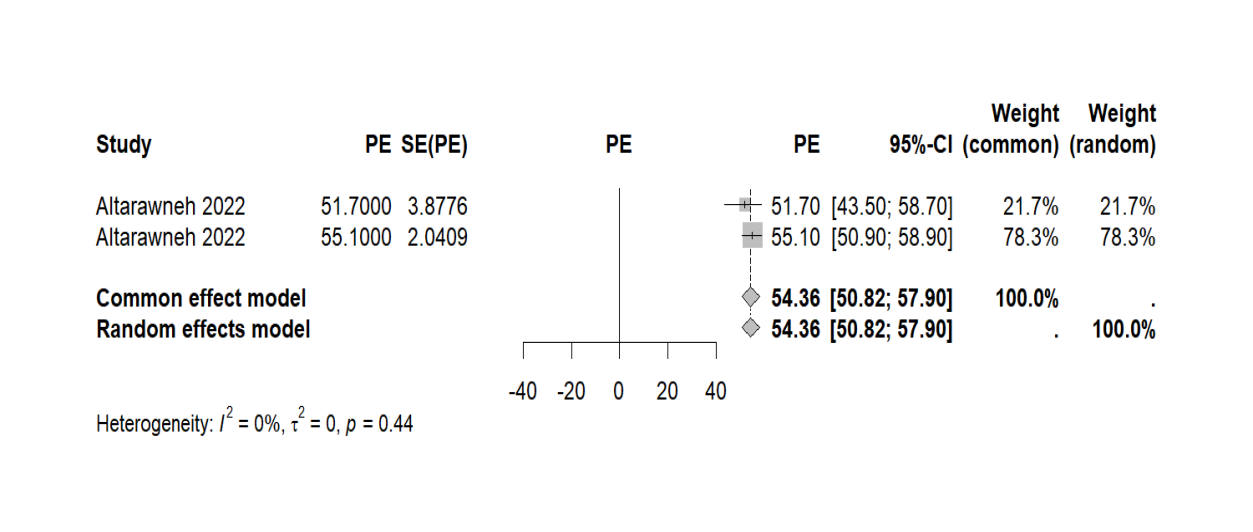


FIGURE H. Effectiveness in mitigating Omicron reinfection after 270-364 days from hybrid immunity (complete vaccination).

# **Forest plots**

## **Analysis on the duration of protection against Omicron reinfection with hybrid immunity (booster vaccination) (Inside the article in Table 4)**


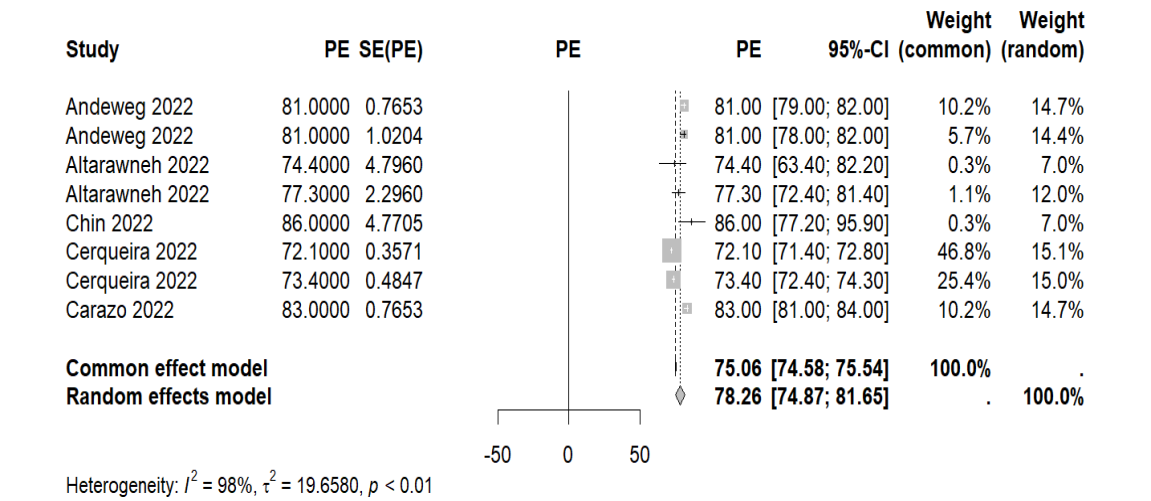


FIGURE A. Effectiveness in mitigating Omicron reinfection after 30-59 days from hybrid immunity (booster vaccination).


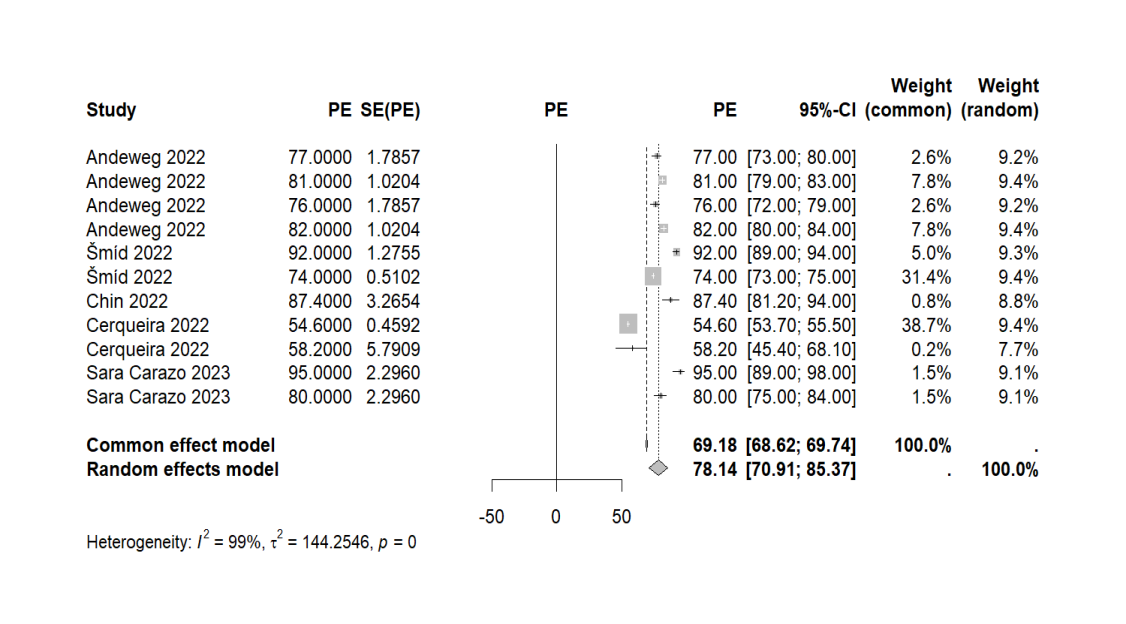


FIGURE B. Effectiveness in mitigating Omicron reinfection after 60-89 days from hybrid immunity (booster vaccination).


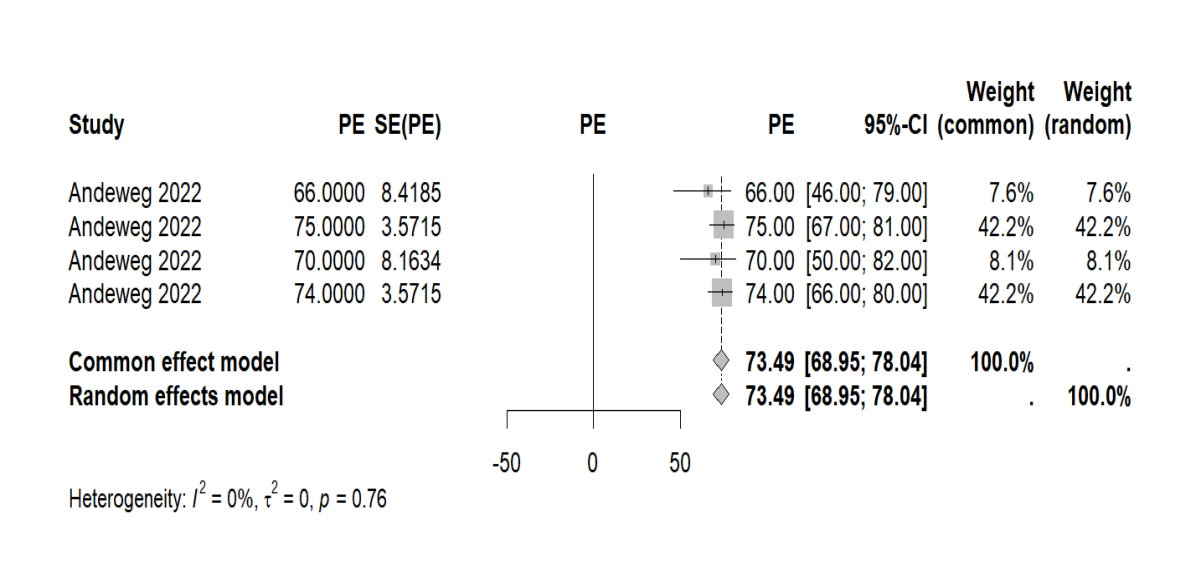


FIGURE C. Effectiveness in mitigating Omicron reinfection after 90-119 days from hybrid immunity (booster vaccination).
