# Supplementary material for: Meta-analysis of hybrid immunity to mitigate the risk of Omicron variant reinfection
Source: Front Public Health. 2024 Aug 26;12:1457266. doi: 10.3389/fpubh.2024.1457266 (PMC11381385; doi:10.3389/fpubh.2024.1457266)
Supplement: Supplementary file 14 [file Table_13.DOCX]

Table 1. Risk analysis of hybrid immunity against reinfection with Omicron variant. (Inside the article in Table 1).

| Exposure group | Control group | Number of articles | Number of studies | Exposure group | |  | Control group | | Pooled OR estimate  and 95%CI | *P* | *I^2^*（%） | *P* | Effect model | *P* for Egger’s test |
| --- | --- | --- | --- | --- | --- | --- | --- | --- | --- | --- | --- | --- | --- | --- |
|  |  |  |  | The number of reinfections | Total number |  | The number of reinfections | Total number |  |  |  |  |  |  |
| Hybrid immunity (incomplete vaccination) | Natural immunity | 10 | 11 | 17285 | 1782524 |  | 187528 | 19535373 | 0.64（0.44-0.93） | 0.027 | 97.0 | <0.001 | random | 0.962 |
| Hybrid immunity (complete vaccination) | Natural immunity | 13 | 25 | 53020 | 5044281 |  | 231588 | 19739066 | 0.58（0.45-0.74） | <0.001 | 99.0 | <0.001 | random | 0.392 |
| Hybrid immunity (booster vaccination) | Natural immunity | 13 | 18 | 28079 | 8769132 |  | 191164 | 19518535 | 0.43（0.34-0.56） | <0.001 | 97.0 | <0.001 | random | 0.126 |
| Hybrid immunity (complete vaccination) | Complete vaccination | 3 | 7 | 5280 | 40416 |  | 67011 | 327551 | 0.35（0.27-0.46） | <0.001 | 97.0 | <0.001 | random |  |
| Hybrid immunity (booster vaccination) | Booster vaccination | 4 | 14 | 3489 | 104802 |  | 159244 | 1252783 | 0.29（0.17-0.47） | <0.001 | 99.0 | <0.001 | random | 0.303 |
| Hybrid immunity (booster vaccination) | Hybrid immunity (complete vaccination) | 9 | 11 | 1671 | 13488 |  | 4663 | 18270 | 0.71（0.61-0.84） | <0.001 | 76.0 | <0.001 | random | 0.806 |
| Hybrid immunity (booster vaccination) | Hybrid immunity (incomplete vaccination) | 9 | 9 | 23315 | 8701929 |  | 1827 | 165726 | 0.50（0.36-0.69） | <0.001 | 92.0 | <0.001 | random |  |
| Hybrid immunity (complete vaccination) | Hybrid immunity (incomplete vaccination) | 9 | 9 | 23404 | 4714338 |  | 2519 | 173648 | 0.69（0.55-0.87） | <0.001 | 93.0 | <0.001 | random |  |

| Table 2. Analysis on durability of protection against Omicron reinfection with hybrid immunity (incomplete vaccination) (days)*. (Inside the article in Table 2). | | | | | | | |  |
| --- | --- | --- | --- | --- | --- | --- | --- | --- |
| Time from incomplete vaccination to reinfection | Number of articles | Number of studies | Pooled PE estimate and 95%CI | *P* | *I^2^*（%） | *P* | Effect model | *P* for Egger’s test |
|  |  |  |  |  |  |  |  |  |
| <60 | 4 | 9 | 66.76%  （58.20%-75.32%） | <0.001 | 76.3 | <0.001 | random |  |
| 60~89 | 2 | 3 | 64.25%  （53.72%-74.77%） | <0.001 | 78.1 | 0.010 | random |  |
| 90~179 | 11 | 35 | 59.50%  （55.56%-63.44%） | <0.001 | 97.8 | <0.001 | random | 0.159 |
| 180~209 | 2 | 7 | 50.72%  （34.12%-67.32%） | <0.001 | 95.1 | <0.001 | random |  |
| 210~269 | 7 | 13 | 42.15%  （32.88%-51.41%） | <0.001 | 98.8 | <0.001 | random | 0.656 |
| 270~364 | 8 | 12 | 37.88%  （28.88%-46.89%） | <0.001 | 98.1 | <0.001 | random | 0.022 |
| 365~639 | 8 | 15 | 33.23%  （23.80%-42.66%） | <0.001 | 97.8 | <0.001 | random | 0.839 |

* The exposed group was hybrid immunity (incomplete vaccination); the control group was never infected and unvaccinated.

Table 3. Analysis on durability of protection against Omicron reinfection with hybrid immunity (complete vaccination) (days)*. (Inside the article in Table 3).

| Time from complete vaccination to reinfection | Number of  articles | Number of studies | Pooled PE Estimate  and 95%CI | *P* | *I^2^*（%） | *P* | Effect model | *P* for Egger’s test |
| --- | --- | --- | --- | --- | --- | --- | --- | --- |
|  |  |  |  |  |  |  |  |  |
|  |  |  |  |  |  |  |  |  |
| 30~59 | 5 | 17 | 76.87%  （69.43%-84.31%） | <0.001 | 99.3 | <0.001 | random | 0.898 |
| 60~89 | 2 | 9 | 76.14%  （65.61%-86.68%） | <0.001 | 99.8 | <0.001 | random |  |
| 90~119 | 2 | 9 | 73.15%  （60.78%-85.53%） | <0.001 | 99.6 | <0.001 | random |  |
| 120~149 | 3 | 9 | 68.68%  （60.91%-76.45%） | <0.001 | 90.9 | <0.001 | random |  |
| 150~179 | 1 | 3 | 64.85%  （57.67%-72.04%） | <0.001 | 0.0 | 0.70 | fixed |  |
| 180~209 | 1 | 4 | 63.08%  （56.15%-70.01%） | <0.001 | 0.0 | 0.801 | fixed |  |
| 210~269 | 3 | 5 | 62.97%  （60.67%-65.26%） | <0.001 | 0.0 | 0.720 | fixed |  |
| 270~364 | 1 | 2 | 54.36%  （50.82%-57.90%） | <0.001 | 0.0 | 0.438 | fixed |  |

* The exposed group was hybrid immunity (complete vaccination); the control group was never infected and unvaccinated.

Table 4. Analysis on durability of protection against Omicron reinfection with hybrid immunity (booster vaccination) (days)*. (Inside the article in Table 4).

| Time from booster vaccination to  reinfection | Number of articles | Number of studies | Pooled PE Estimate  and 95%CI | *P* | *I^2^*（%） | *P* | Effect model | *P* for Egger’s test |
| --- | --- | --- | --- | --- | --- | --- | --- | --- |
|  |  |  |  |  |  |  |  |  |
|  |  |  |  |  |  |  |  |  |
| 30~59 | 5 | 8 | 78.26%  （74.87%-81.65%） | <0.001 | 97.7 | <0.001 | random |  |
| 60~89 | 5 | 11 | 78.14%  （70.91%-85.37%） | <0.001 | 99.5 | <0.001 | random | 0.079 |
| 90~119 | 1 | 4 | 73.49%  （68.95%-78.04%） | <0.001 | 0.0 | 0.759 | fixed |  |

*The exposed group was hybrid immunity (booster vaccination); the control group was never infected and unvaccinated.
